# Identification of (Z)-2-benzylidene-dihydroimidazothiazolone derivatives as tyrosinase inhibitors: Anti-melanogenic effects and *in silico* studies

**DOI:** 10.1016/j.csbj.2022.02.007

**Published:** 2022-02-12

**Authors:** Heejeong Choi, Il Young Ryu, Inkyu Choi, Sultan Ullah, Hee Jin Jung, Yujin Park, YeJi Hwang, Yeongmu Jeong, Sojeong Hong, Pusoon Chun, Hae Young Chung, Hyung Ryong Moon

**Affiliations:** a College of Pharmacy, Pusan National University, Busan 46241, South Korea; b Department of Molecular Medicine, The Scripps Research Institute, FL 33458, USA; c College of Pharmacy and Inje Institute of Pharmaceutical Sciences and Research, Inje University, Gimhae, Gyeongnam 50834, South Korea

**Keywords:** (Z)-2-Benzylidene-dihydroimidazothiazolone, Docking simulation, Kojic acid, Tyrosinase inhibitor, Anti-melanogenesis, DHIT template

## Abstract

As part of our continuous search for novel tyrosinase inhibitors, we designed 5,6-dihydroimindazo[2,1-*b*]thiazol-3(2*H*)-one (DHIT) derivatives based on the structure of MHY773; a potent tyrosinase inhibitor with a 2-iminothiazolidin-4-one template. Of the 11 DHIT derivatives synthesized using a Knoevenagel condensation, three DHIT derivatives **1a** (IC_50_ = 36.14 ± 3.90 μM), **1b** (IC_50_ = 0.88 ± 0.91 μM), and **1f** (IC_50_ = 17.10 ± 1.01 μM) inhibited mushroom tyrosinase more than kojic acid (IC_50_ = 84.41 ± 2.87 μM). Notably, compound **1b** inhibited mushroom tyrosinase around 100- and 3.3-fold more potently than kojic acid and MHY773, respectively. Lineweaver-Burk plots demonstrated that compounds **1b** and **1f** competitively inhibited mushroom tyrosinase, and *in silico* docking results supported our kinetic results and indicated that these two compounds bind more strongly to the active site of tyrosinase than kojic acid. Docking simulation results using a human tyrosinase homology model confirmed the abilities of **1b** and **1f** to strongly inhibit human tyrosinase. B16F10 murine melanoma cells were used to investigate whether these two compounds display tyrosinase inhibitory activities and anti-melanogenesis effects in cells. Both compounds were found to significantly and dose-dependently inhibit cellular tyrosinase activity and intracellular and extracellular melanin production more potently than kojic acid. The similarities observed between the cellular tyrosinase and melanogenesis inhibitory effects of **1b** and **1f** suggest their observed anti-melanogenic effects were due to tyrosinase inhibition. These results indicate that compounds **1b** and **1f**, which possess the DHIT template, are promising candidates as anti-browning agents and therapeutic agents for hyperpigmentation disorders.

## Highlights

• Of the synthesized 11 DHIT derivatives, three derivatives **1a**, **1b** and **1c** exhibited stronger mushroom tyrosinase inhibition than kojic acid, a representative tyrosinase inhibitor.
• The derivative **1b** showed roughly 100- and 3.3-fold more potent tyrosinase inhibitory activity than kojic acid and MHY773, respectively.
• Kinetic analyses using Lineweaver-Burk plots demonstrated that **1b** and **1f** are competitive inhibitors and *in silico* docking results using mushroom tyrosinase supported the kinetic results.
• In B16F10 cells, **1b** and **1f** inhibited cellular tyrosinase activity and intra- and extracellular melanin productions more potently than kojic acid without perceptible cytotoxicity.
• These results suggest that **1b** and **1f** bearing the DHIT template can be promising candidates for therapeutic agents for hyperpigmentation disorders or anti-browning agents.

## Graphical abstract

**Figure:**
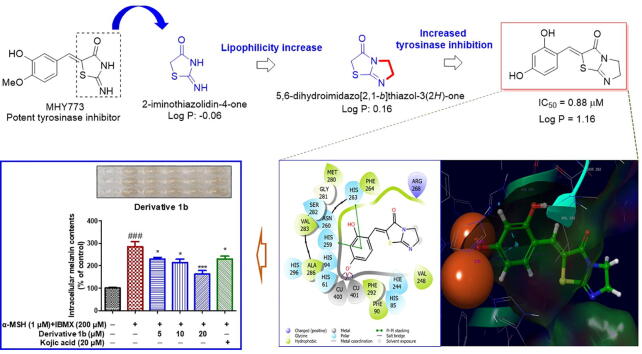

## 1 Introduction

Tyrosinase (TYR, EC 1.14.18.1) is the rate-limiting enzyme in melanogenesis and is found in mammals, plants, fungi, and bacteria. Human tyrosinase (*h*TYR), which belongs to the mammalian tyrosinase family, is a type 3 copper-containing metalloenzyme and a glycoprotein comprised of 529 amino acids. At its active site, two magnetically bound copper ions (Cu_a_ and Cu_b_) are linked by a hydroxo ligand in the *met* state and coordinated with six histidine residues [1]. The primary function of *h*TYR is to oxidize L-tyrosine to L-DOPA by acting as monophenolase and subsequently to convert L-DOPA to dopaquinone by acting as a diphenolase [2]. This double oxidation process leads to the formation of melanin. Two other enzymes, tyrosinase-related proteins 1 and 2 (*h*TYRP1 and *h*TYRP2) are also known to participate in the production of melanin. The final products of melanogenesis are eumelanin (a dark brown to black pigment) and pheomelanin (a yellow/red pigment) [3]. Melanogenesis occurs in melanocytes, a specialized type of dendritic cell, located in skin, hair bulbs, and eyes. At the subcellular level, melanins are stored in melanosomes and importantly protect skin against UV radiation and free radicals. However, abnormal productions of melanins can cause pathological conditions, which include melasma, lentigo, congenital melanocytic naevi, erythormelanosis follicularis faciei et colli, post-inflammatory hyperpigmentation, and erythema dyschromicum perstans [4]. Because *h*TYR catalyzes the first two steps of melanogenesis, most of the efforts made to suppress or reduce melanin production have been directed at the development of effective TYR inhibitors. This strategy has proven to be effective and a strong correlation has been demonstrated between TYR inhibition and levels of melanin generated [5]. Thus, *h*TYR is considered an attractive target for reducing melanogenesis but is rarely used due to the high price of *h*TYR and the difficulties associated with their productions in stable forms [5]. To assay TYR inhibitory activity of potential tyrosinase inhibitors, mushroom (*Agaricus bisporus*) tyrosinase (*ab*TYR) is widely used as a study model because of its availability, low cost and reliable results.

Thousands of TYR inhibitors have been reported [6], [7], [8], [9], [10], [11], and some, such as kojic acid [12], hydroquinone, monobenzyl hydroquinone, arbutin [13], [14], [15], [16], [17], salicylhydroxamic acid [18], azelaic acid [19], rucinol [20], phenylethyl resorcinol [21], thiazolyl resorcinols [22], and corticosteroids [18], [19], exhibit excellent anti-melanogenic effects in cell-based assays [23], [24], [25], [26], [27], [28], [29], [30], [31], [32], [33], [34], [35]. However, many of these inhibitors have been reported to have severe side effects, such as skin cancer, irreversible depigmentation, and dermatitis, in animal and human models [36]. Hydroquinone and kojic acid are banned in most countries due to their carcinogenic [37], nephrotoxic [38], melanocytoxic [39], and genotoxic [40] effects. Arbutin (a natural tyrosinase inhibitor) has lesser side effects but has stability issues, for example, it is hydrolyzed to hydroquinone and D-glucose by thermodegradation at 20 °C and skin microflora [41], [42] Accordingly, research efforts are aimed at identifying safer, more potent stable tyrosinase inhibitors.

**Fig. 1:**
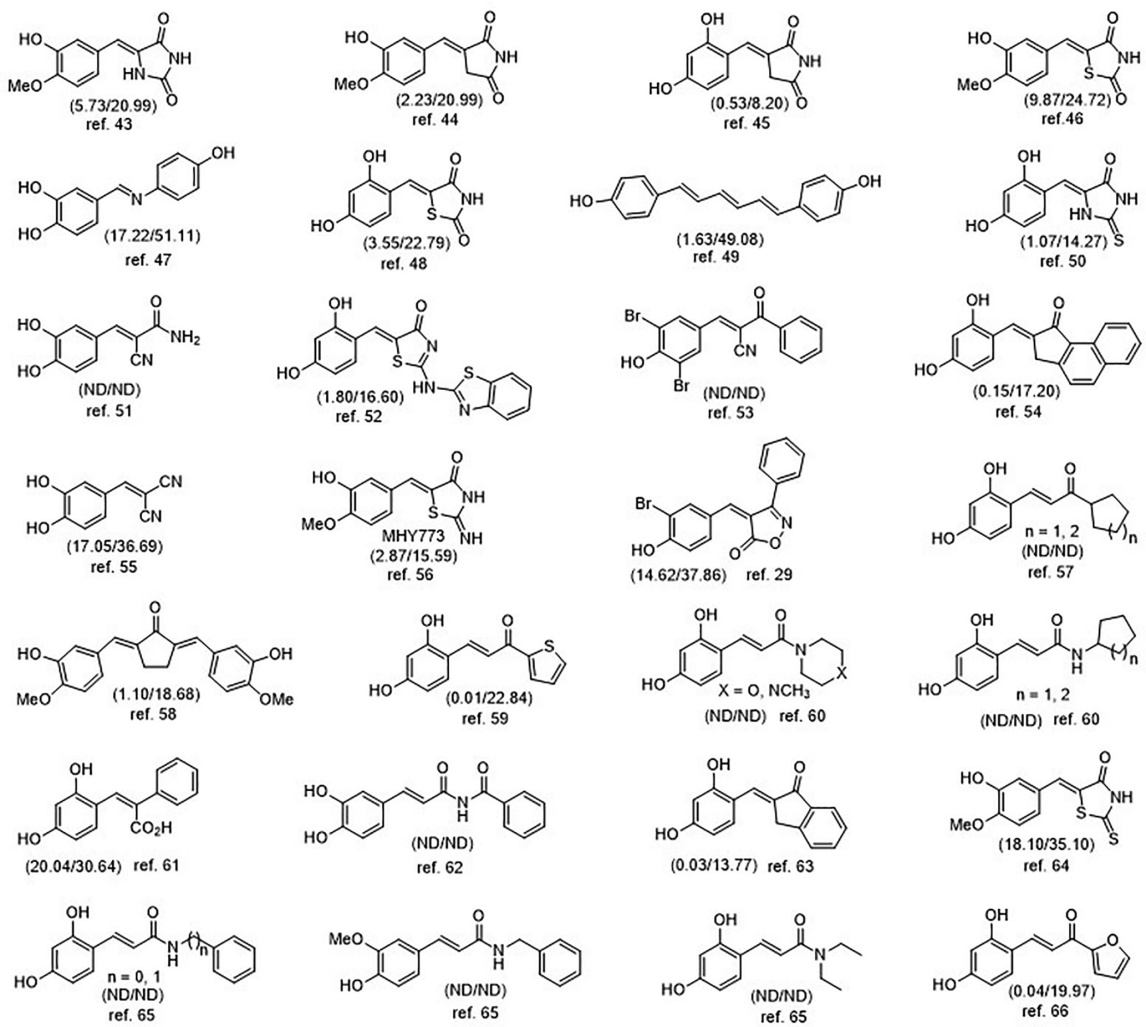
Substituted benzylidene tyrosinase inhibitors with a variety of templates. The values in parenthesis represent IC_50_ values (μM) of the corresponding compound and kojic acid, respectively. ND means ‘not determined’.

During our studies on this topic over past decades, we have identified a number of benzylidene derivatives with the β-phenyl-α,β-unsaturated carbonyl scaffold more potently inhibit tyrosinase than kojic acid, a representative tyrosinase inhibitor (Fig. 1) [43], [44], [45], [46], [47], [48], [49], [50], [51], [52], [53], [54], [55], [56], [29], [57], [58], [59], [60], [61], [62], [63], [64], [65], [66].

**Fig. 2:**
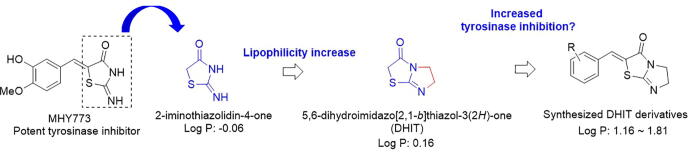
Rationale for the design of DHIT derivatives.

Celecoxib and rofecoxib are nonsteroidal anti-inflammatory drugs. Although rofecoxib was withdrawn from the market due to increased heart attack risk, rofecoxib with a higher log P value penetrated the blood–brain barrier better than celecoxib. As such, log P values are closely related to brain penetration of drugs and absorption of drugs into the skin and gastrointestinal tract. Previously, we synthesized (Z)-5-(3-hydroxy-4-methoxybenzylidene)-2-iminothiazolidin-4-one (MHY773, Fig. 1), which possesses a 2-iminothiazolidin-4-one template, and found this compound potently inhibited mushroom tyrosinase (IC_50_ = 2.87 μM for monophenolase and 8.06 μM for diphenolase) and melanogenesis in B16F10 melanoma cells [56]. The effectiveness of whitening agents depend on how well they access melanocytes in the epidermal basal layer, and skin permeability is known to be closely related to lipophilicity. As shown in Fig. 2, the chemical structure of 5,6-dihydroimindazo[2,1-*b*]thiazol-3(2*H*)-one (DHIT) is similar to that of 2-iminothiazolidin-4-one, which is more lipophilic, and thus, we expected derivatives with the DHIT template to potently inhibit tyrosinase and melanin production. Thus, we selected as a novel core template for tyrosinase inhibitors and designed and synthesized 11 DHIT derivatives with a 2-benzylidene group bearing a variety of substituents. These 11 derivatives were evaluated for mushroom tyrosinase inhibitory activity at the enzyme level and for tyrosinase activity and melanogenesis in cell-based systems. The underlying mechanisms involved were investigated using Lineweaver-Burk double reciprocal plots, and to investigate the possibility that these derivatives inhibit human tyrosinase, a homologous model for human tyrosinase was prepared and docking simulations were performed.

## 2 Results and discussion

### 2.1 Chemistry

**Scheme 1:**
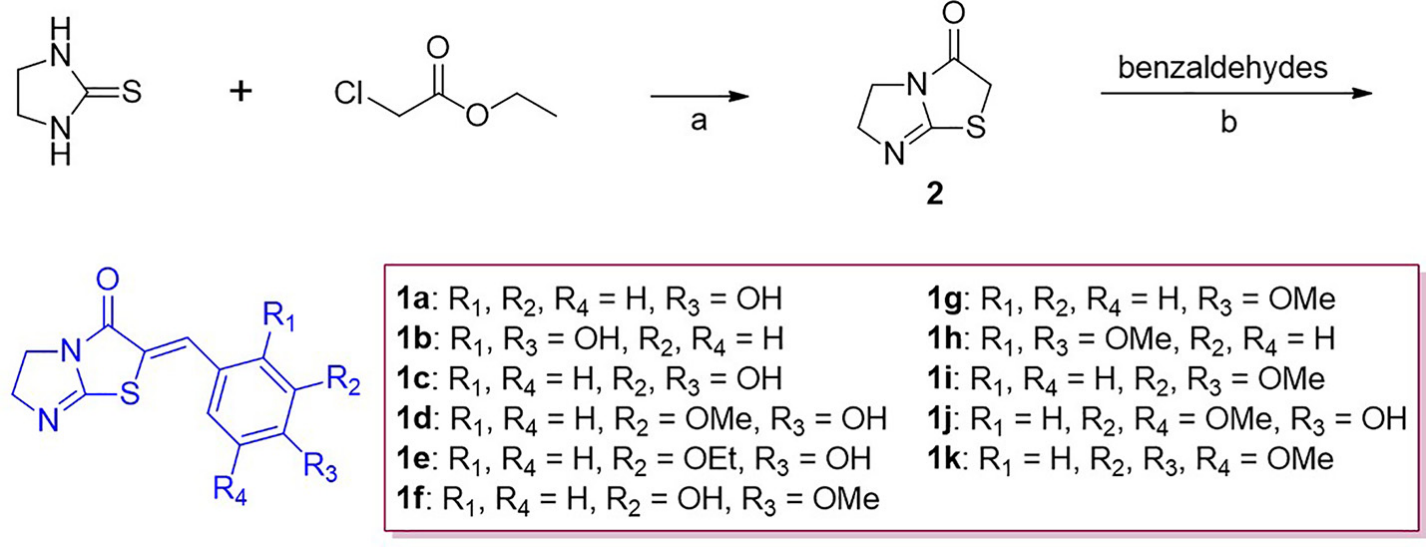
Synthetic scheme for (*Z*)-2-(substituted benzylidene)-5,6-dihydroimidazo[2,1-*b*]thiazol-3(2*H*)-one derivatives. Reagents and conditions: (a) NaOAc, EtOH, reflux, 21 h, 72%; (b) NaOAc, acetic acid, 80 °C, 2 – 12 h, 37 – 88%.

First, 5,6-dihydroimidazo[2,1-*b*]thiazol-3(2*H*)-one (**2**) [67], the DHIT template, was prepared by reacting 2-thioimidazolidine with ethyl chloroacetate in the presence of sodium acetate (yield 72%) (Scheme 1) [68]. Reaction of **2** with benzaldehydes under Knoevenagel condensation conditions using sodium acetate/acetic acid gave benzylidene derivatives **1a** – **1 k** (all bearing the DHIT template) in yields of 37 to 88% [69]. To examine the effects of substituents of the benzylidene moiety on tyrosinase inhibition, various benzaldehydes bearing substituents such as a hydroxyl, a methoxyl, and/or an ethoxyl group were condensed with **2**. A total of 11 derivatives with the DHIT template were synthesized.

**Fig. 3:**
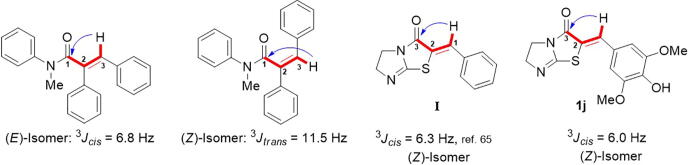
Relationships between the geometry of the α -carbonyl C = C double bond and C,H-spin-coupling constants over three bonds as indicated by the arrow.

The structures of the eleven DHIT derivatives were determined by ^1^H and ^13^C NMR and mass (MS) spectroscopy. The double bond geometries of all 11 derivatives (**1a** – **1 k**) were assigned to the (*Z*)-configuration based on vicinal ^1^H, ^13^C-coupling constants (^3^*J*) in proton-coupled ^13^C NMR spectra. According to a report by Nair et al. [70], different vicinal ^1^H, ^13^C-coupling constants in proton-coupled ^13^C NMR spectra are observed for geometric isomers of a variety of trisubstituted α ,β-unsaturated carbonyl compounds, including 5-membered and 6-membered exocyclic methylene carbonyl compounds. As depicted in Fig. 3, vicinal coupling constants between the amide carbonyl C-atom C(1) and the olefinic H-atom at C(3) in proton-coupled ^13^C NMR spectra depended on double bond stereochemistry: (*Z*)-isomer: ^3^*J_trans_* = 11.5 Hz, and (*E*)-isomer: ^3^*J_cis_* = 6.8 Hz. Generally, ^3^*J_cis_* values range from 3.6 to 7.0 Hz, while the range of ^3^*J_trans_* is roughly twice as large (typically ≥ 10 Hz) [71]. For compound **I** (Fig. 3), the amide carbonyl carbon had a vicinal ^1^H, ^13^C-coupling constant of 6.3 Hz (^3^*J*_(C(3),_ *_H_*_-C(1))_) [71]. The ^13^C NMR of compound **1b** was measured in proton-coupled ^13^C mode, and the ^3^*J* value of C3 in **1j** was 6.0 Hz (Fig. S30 in Supplementary data), suggesting a (*Z*)-configuration.

### 2.2 Tyrosinase inhibition - Kinetics, mechanism, and *in silico* and in vitro studies

#### 2.2.1 Mushroom tyrosinase inhibition

The anti-tyrosinase efficacies of the 11 synthesized DHIT derivatives **1a** – **1 k** against mushroom tyrosinase (*m*TYR) were investigated as we previously described for the evaluation of *m*TYR inhibitory activity [65]. Table 1 summarizes % inhibition results for **1a** – **1 k** at a concentration of 25 μM. Kojic acid was used as a positive control, as is typical for tyrosinase inhibitor evaluations. Compounds **1 g**, **1 h**, **1i**, and **1 k** with no hydroxyl substituent showed low or no *m*TYR inhibitory activity, whereas compounds **1a**, **1b**, and **1f** inhibited *m*TYR more than kojic acid (30% inhibition) at 25 μM. Compound **1a** (40% inhibition) with a 4-hydroxyl substituent on the phenyl ring inhibited *m*TYR slightly more than kojic acid. However, compound **1f** (73% inhibition) with a 3-hydroxy-4-methoxyl substituent on the phenyl ring inhibited *m*TYR more than compound **1a**. Compound **1b** (96% inhibition) with a hydroxyl at the 2- and 4-positions of the phenyl ring had greatest inhibitory effect. Considering the potent *m*TYR inhibitory activities of compounds **1a** and **1b**, it seemed that the presence of a 4-hydroxyl on the phenyl ring markedly increased *m*TYR inhibition, but compounds **1d** and **1e**, which also possessed a 4-hydroxyl exhibited only low mushroom tyrosinase inhibitory activity (7 and 3%, respectively). These results indicate that the presence of a 4-hydroxyl on the phenyl rings of DHIT derivatives can markedly influence *m*TYR inhibition, but that the additional presence of a 3-alkoxyl (3-methoxyl in **1d**, or 3-ethoxyl in **1e**) substituent or a 3,5-dialkoxyl (3,5-dimethoxyl in **1j**) suppresses 4-hydroxyl-induced decreases in *m*TYR activity. Compounds **1b** and **1c** had a 4-hydroxyl substituent and at a different position on the phenyl ring had an extra hydroxyl substituent. Interestingly, these compounds inhibited *m*TYR to different extents, that is, compound **1b** inhibited *m*TYR by 96% whereas compound **1c** inhibited *m*TYR by only 18%. These results indicate that the position of an extra hydroxyl substituent on the 4-hydroxyphenyl ring can greatly influence *m*TYR inhibition. Our accumulated SAR data on tyrosinase inhibitors bearing a β -phenyl- α, β -unsaturated carbonyl motif also suggests that compounds with a 2,4-dihydroxyl or 3-hydroxy-4-methoxyl substituent on the β -phenyl ring potently inhibit *m*TYR (Fig. 1). In accord with our collated SAR results, compound **1b** with a β −2,4-dihydroxyphenyl group and compound **1f** with a β −3-hydroxy-4-methoxyphenyl group were found to most potently inhibit *m*TYR. According to the cumulative docking simulation results, the 2,4-dihydroxyl substituent on the β -phenyl ring contributes to strong binding to tyrosinase through hydrogen bonding at the active site, implying that the 2,4-dihydroxyl substituent plays an important role in tyrosinase inhibition. In some compounds [53], [58], [61], [62], [64], [66], both hydroxyl groups of the 2,4-dihydroxyl substituent participate in hydrogen bonding as hydrogen bond donors, and in some compounds [49], [60], [61], [67] only one of the two hydroxyls participates in hydrogen bonding as a hydrogen bond donor.

**Table 1:** Substitution patterns and mushroom tyrosinase inhibitions after treatment with (*Z*)-2-(substituted benzylidene)-5,6-dihydroimidazo[2,1-*b*]thiazol-3(2*H*)-one derivatives **1a** – **1 k** or kojic acid, and log P values.

Compound	R_1_	R_2_	R_3_	R_4_	Tyrosinase inhibition (%)	IC_50_ (μM)	Synthetic yield (%)	*^a^*Log P
**1a**	H	H	OH	H	40.89 ± 0.68	36.14 ± 3.90	68	1.55
**1b**	OH	H	OH	H	96.69 ± 0.01	0.88 ± 0.91	59	1.16
**1c**	H	OH	OH	H	18.96 ± 0.44	>100	74	1.16
**1d**	H	OMe	OH	H	7.83 ± 2.29	>100	37	1.42
**1e**	H	OEt	OH	H	3.90 ± 2.65	>100	72	1.76
**1f**	H	OH	OMe	H	73.73 ± 1.35	17.10 ± 1.01	63	1.42
**1 g**	H	H	OMe	H	9.32 ± 1.08	>100	65	1.81
**1 h**	OMe	H	OMe	H	13.41 ± 0.93	>100	73	1.69
**1i**	H	OMe	OMe	H	*^b^*NI	>100	88	1.69
**1j**	H	OMe	OH	OMe	24.43 ± 1.50	>100	55	1.30
**1 k**	H	OMe	OMe	OMe	NI	>100	78	1.56
*^c^*KA					30.26 ± 0.55	84.41 ± 2.87		−2.45

Tyrosinase inhibition experiments were conducted using synthesized derivatives or kojic acid at a concentration of 25 μM. *^a^*Log P values were obtained using ChemDraw Ultra 12.0. *^b^*NI means no inhibition. *^c^*KA means kojic acid.

Because DHIT derivatives potently inhibited mushroom tyrosinase, we investigated the IC_50_ values of the synthesized DHIT derivatives. The three active derivatives **1a**, **1b**, and **1f** dose-dependently inhibited *m*TYR (data not shown). The IC_50_ value of kojic acid, the positive control, was 84.41 μM, while those of DHIT derivatives **1a** and **1f** were 36.14 and 17.10 μM, respectively, indicating that they were 2.3- and 4.9-fold stronger tyrosinase inhibitors than kojic acid. Compound **1b** inhibited *m*TYR most with an IC_50_ value of 0.88 μM, which showed it inhibited *m*TYR 100-fold more than kojic acid. The other DHIT derivatives had IC_50_ values above 100 μM.

Log P values of the synthesized DHIT derivatives were obtained using ChemDraw Ultra 12.0. As indicated in Table 1, DHIT derivatives had log P values ranging from 1.16 to 1.81, which showed they were more lipophilic than the corresponding derivatives with the 2-iminothiazolidin-4-one template.

#### 2.2.2 Determination of the inhibitory mechanism by enzyme kinetics

**Fig. 4:**
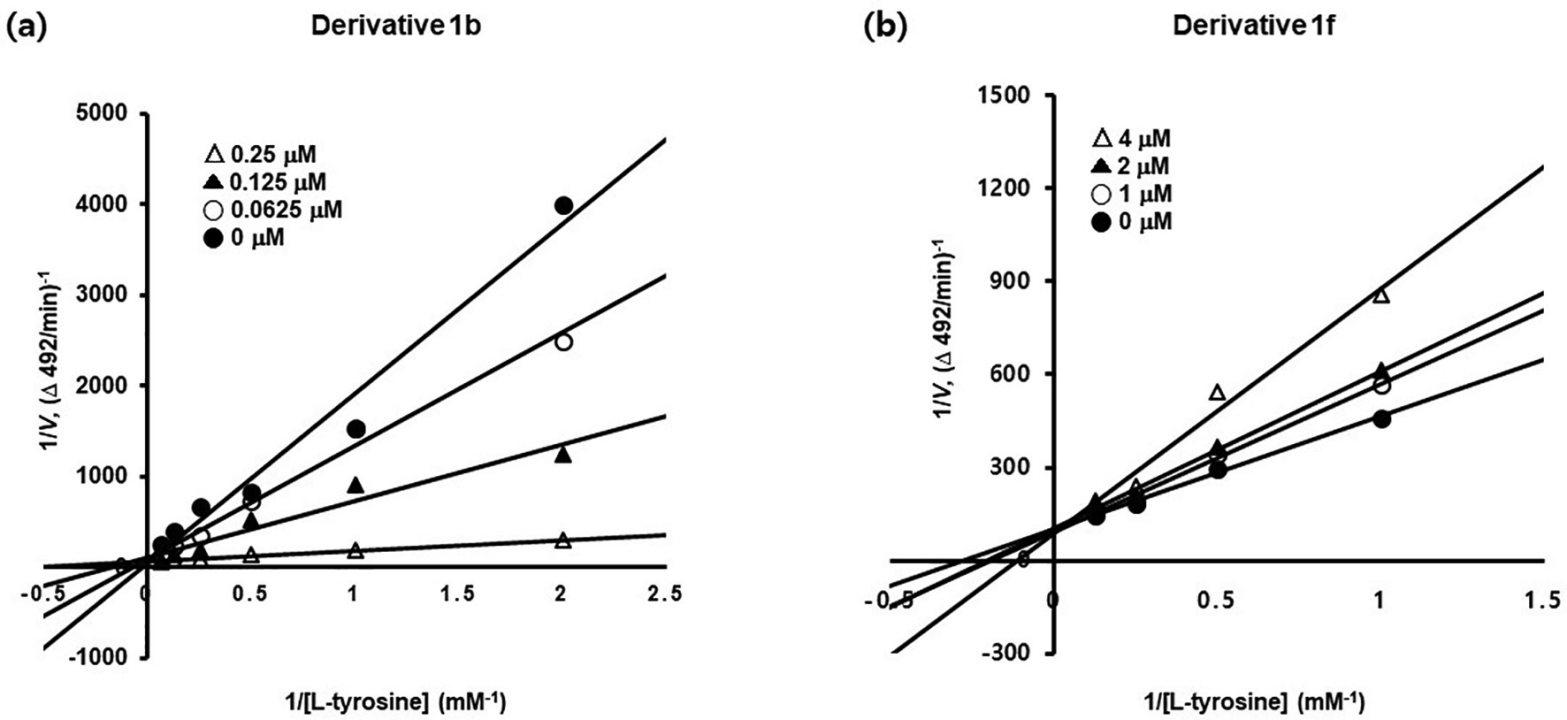
Enzyme inhibition kinetic studies of DHIT derivatives **1b** (a) and **1f** (b) against mushroom tyrosinase. Lineweaver-Burk plots for the inhibition of *m*TYR were obtained at different concentrations of **1b** and **1f** (0 µM (closed circle), 0.0625 µM (open circle), 0.125 µM (closed triangle) and 0.25 µM (open triangle) for **1b**; and 0 µM (closed circle), 1 µM (open circle), 2 µM (closed triangle) and 4 µM (open triangle) for **1f**) in the presence of different concentrations of l-tyrosine (0.5, 1, 2, 4, 8, or 16 mM for **1b** and 1, 2, 4, or 8 mM for **1f**).

Since compounds **1b** and **1f** most potently inhibited *m*TYR, they were subjected to kinetic study. Lineweaver-Burk double reciprocal plots were used to determine their inhibitory modes of action. Kinetic analyses were carried out using four concentrations of DHIT derivatives **1b** (0, 0.0625, 0.125, or 0.25 μM) and **1f** (0, 1, 2, or 4 μM) in the presence of different concentrations of L-tyrosine (0.5, 1, 2, 4, 8, or 16 mM for **1b** and 1, 2, 4, or 8 mM for **1f**). Results are summarized in Fig. 4. Lineweaver-Burk double reciprocal plots produced four different lines with different slopes, corresponding to the four concentrations, for each derivative. In each case, the four different lines converged at one point on the y-axis, indicating that the V_max_ values of the two compounds (0.0126 mM/min for **1b** and 0.0103 mM/min for **1f**) were independent of concentration. In addition, the results showed that the K_M_ values of **1b** and **1f** increased in a concentration-dependent manner, which suggested that both bind to the same binding pocket as L-tyrosine and competitively inhibit *m*TYR. K_M_ values were 1.515, 7.846, 15.740, and 23.586 mM at 0, 0.0625, 0.125, and 0.25 mM of **1b**, and 3.758, 4.892, 5.206, and 8.127 mM at 0, 1, 2, and 4 μM of **1f**, respectively. Furthermore, the kinetic studies showed that K_i_ values were 1.50 × 10^−8^, 1.33 × 10^−8^, and 1.72 × 10^−8^ M at 0.0625, 0.125, 0.25 μM of **1b**, and 3.31 × 10^−6^, 5.19 × 10^−6^, and 3.44 × 10^−6^ M at 1, 2, and 4 μM of **1f**, respectively. The binding abilities of compounds **1b** and **1f** to the active site of tyrosinase were also supported by *in silico* docking simulation results (Figs. 5 and 6).

#### 2.2.3 In silico studies of compounds **1b** and **1f** using the crystal structure of mTYR and a hTYR homology model

A docking study was performed using Schrodinger suite (release 2021–1) to further investigate the tyrosinase enzyme inhibitory effects of compounds **1b** and **1f**. Both compounds potently inhibited *m*TYR in the mushroom tyrosinase assay (Table 1), and kinetic studies confirmed the competitive nature of these inhibitions (Fig. 4). To determine whether **1b** and **1f** bind directly to the active sites of *m*TYR and *h*TYR, we docked compounds **1b** and **1f** to the crystal structure of *m*TYR and to a prepared human tyrosinase homology model, respectively, and compared the results with those obtained with kojic acid.

##### 2.2.3.1 Docking studies of derivatives **1b** or **1f** or kojic acid with mTYR

The crystal structure of *m*TYR (*Agaricus bisporus* tyrosinase, PDB ID: 2Y9X) was imported from the Protein Data Bank and docked against **1b**, **1f**, and kojic acid. Interactions between these three compounds and *m*TYR are shown in Fig. 5a in 2D and 3D. Docking results showed **1b** and **1f** occupied the same binding site as kojic acid. To further examine the natures of these interactions we considered pi-pi stacking, hydrophobic interactions, hydrogen bonding, and metal coordination. Kojic acid formed one hydrogen bond with the amino acid residue Met280 using the 5-hydroxyl group of its 4-pyrone ring at a distance of 2.20 Å. In addition, the 2-hydroxymethyl group of its 4-pyrone ring coordinated with Cu401 at a distance of 2.36 Å, and the 4-pyrone ring interacted with His263 by pi-pi stacking, and also with Val283 hydrophobically. Based on these interactions, the recorded docking score for kojic acid was − 4.4 kcal/mol. Interestingly, the binding interactions between *m*TYR and **1b** and kojic acid were closely related. Like kojic acid, the 4-hydroxyl group of the phenyl ring of **1b** also coordinated with Cu401, although notably, the 4-hydroxyl group of the phenyl ring of **1b** also coordinated with Cu400 and formed salt bridges with Cu400 and Cu401 at distances of 2.28 and 2.32 Å, respectively. However, unlike kojic acid, **1b** did not form hydrogen bonds though it did form two pi-pi stacking interactions with His259 and His263. In addition, **1b** interacted hydrophobically with Phe264, Met280, and Val283. Due to these interactions, **1b** had a docking score of − 6.7 kcal/mol, that is, docking analysis indicated it binds to the active site of tyrosinase much more strongly than kojic acid. The 3D structures in Fig. 5 also show that in its bound position **1b** is located closer to the two copper ions than kojic acid. On the other hand, **1f**, unlike kojic acid and **1b**, did not coordinate with copper ions or form a salt bridge, and made one hydrogen bond with Asn260 using the 3-hydroxyl group on its phenyl ring. Interestingly, **1f** interacted hydrophilically with His85, Hie244 (a protonated histidine form), His259, Asn260, and His263, and achieved a docking score of − 4.6 kcal/mol, that is, a score intermediates between those of kojic acid and compound **1b**. These results, which are in line with our kinetic study results, suggest that the strong *m*TYR inhibitory activities of **1b** and **1f** are due to strong binding with the active site of *m*TYR.

**Fig. 5a:**
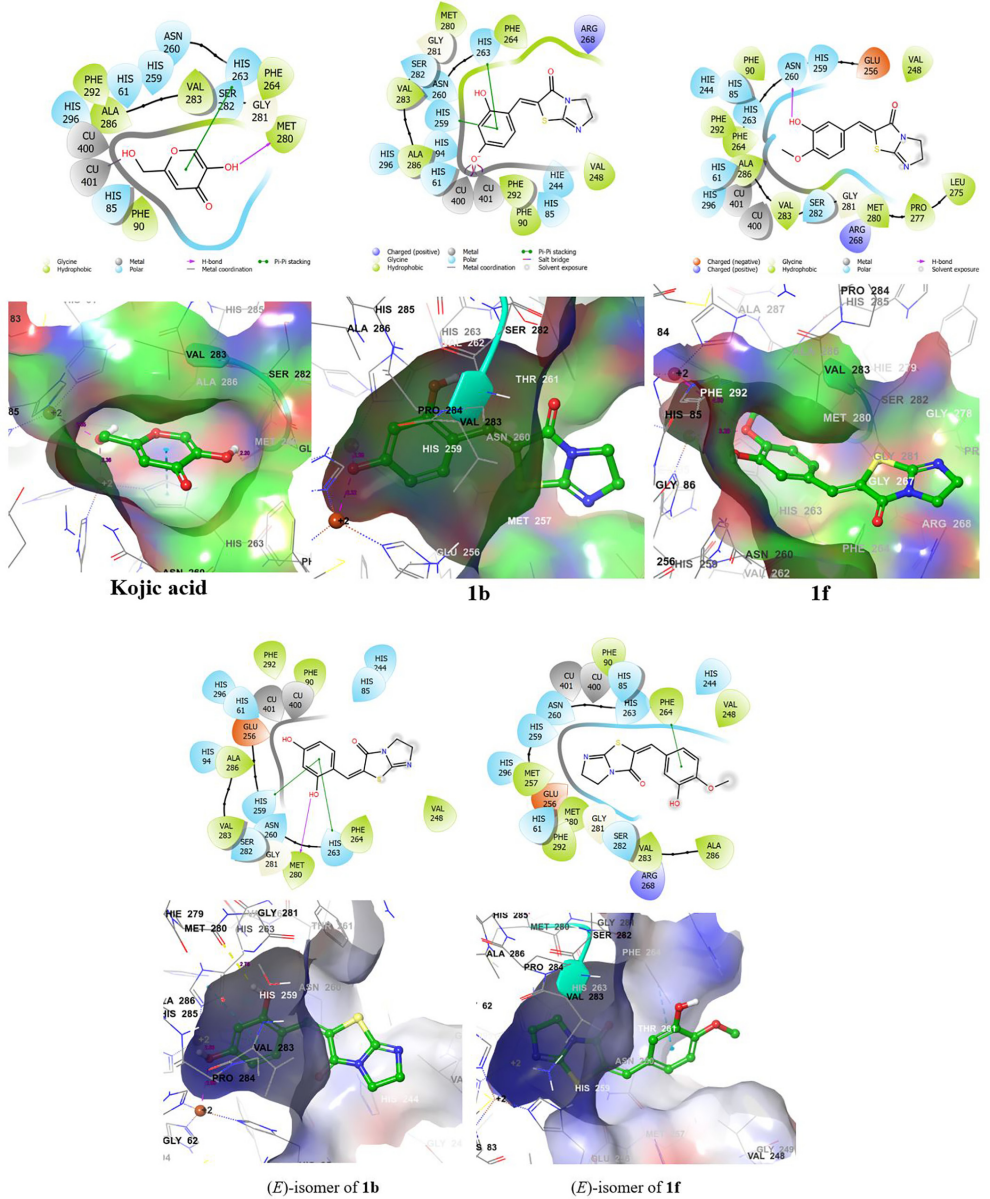
Docking studies on derivatives **1b** or **1f** or kojic acid using Schrödinger suite. *m*TYR (*Agaricus bisporus* tyrosinase, PDB ID = 2Y9X) was used as the tyrosinase for docking simulations. Pharmacophore results for **1b**, **1f**, and kojic acid are represented in 2D and 3D.

We also performed docking studies on the (*E*)-isomers of compounds **1b** and **1f** to determine whether the geometry of the compounds plays a role in the protein-binding interactions at the active site of tyrosinase, as shown in Fig. 5b. Surprisingly, both compounds still occupied the active site of the tyrosinase enzyme, but showed lower docking scores compared to the (*Z*)-isomers, **1b** and **1f**. The lower docking score of the (*E*)-isomer of **1b** may be due to the loss of salt bridges with copper ions. Also, the (*E*)-isomer of compound **1f** showed reverse conformation to that of the (*Z*)-isomer in the active site of tyrosinase enzyme. These docking results suggest that only the (*Z*)-isomers of **1b** and **1f** strongly inhibit the activity of the tyrosinase enzyme.

**Fig. 5b:**
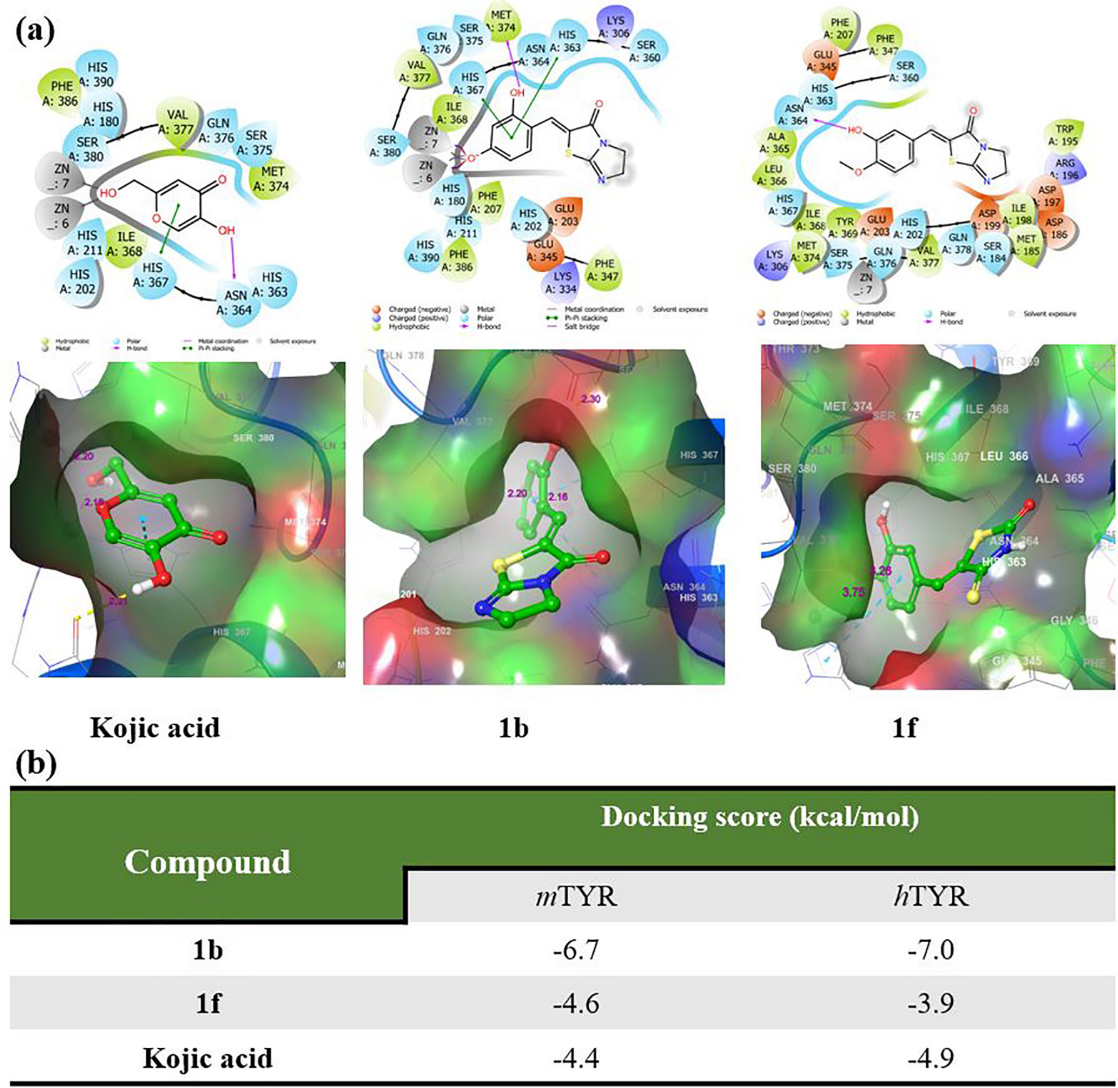
Docking studies on the (*E*)-isomers of **1b** and **1f** using Schrödinger suite. *m*TYR (*Agaricus bisporus* tyrosinase, PDB ID = 2Y9X) was used as the tyrosinase for docking simulations. Pharmacophore results for **1b** and **1f** are represented in 2D and 3D.

##### 2.2.3.2 Docking studies of derivatives **1b** or **1f** or kojic acid with a hTYR homology model

**Fig. 6:**
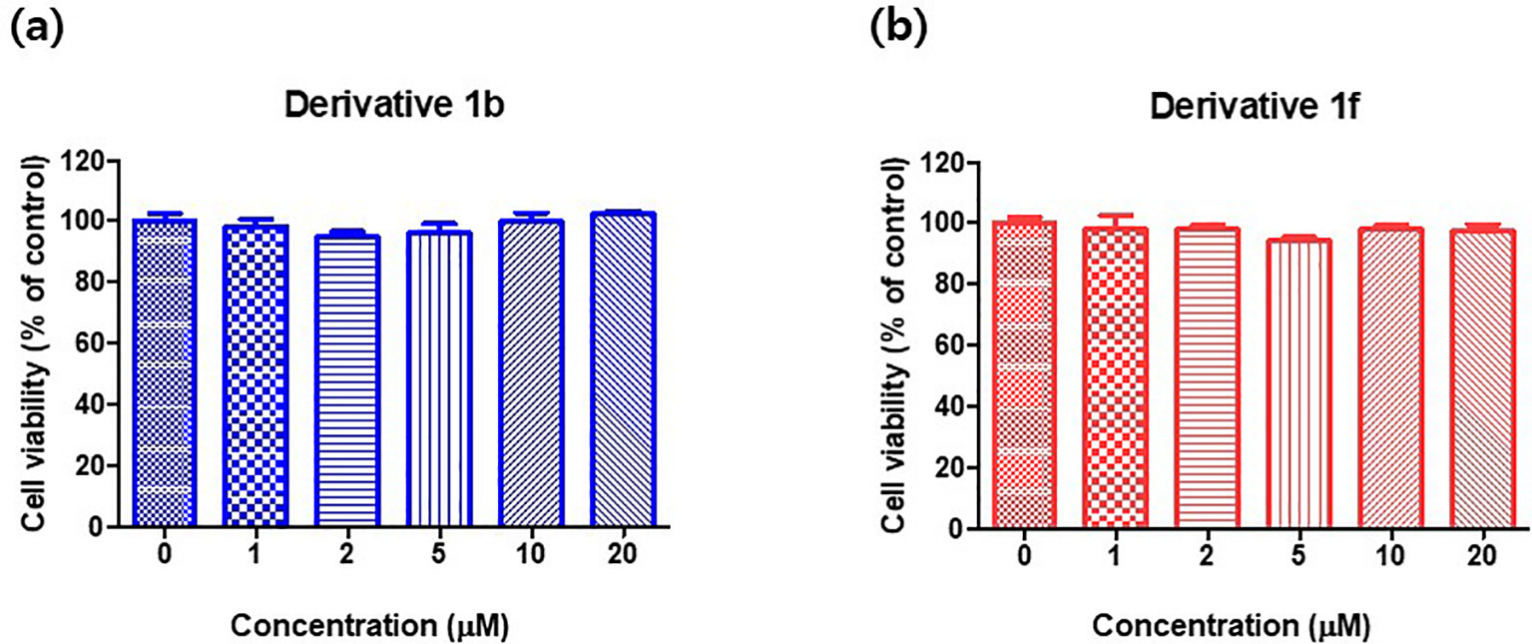
Expected binding modes and docking scores of the DHIT derivatives **1b** or **1f** or kojic acid as determined using the *h*TYR homology model and Schrödinger suite. (a) 2D and 3D representations of **1b**, **1f**, and kojic acid, and (b) the binding affinities of **1b**, **1f**, and kojic acid for mushroom tyrosinase (PDB: 2Y9X) and the human tyrosinase homology model.

After confirming direct binding between **1b** or **1f** and *m*TYR we performed docking simulations using a *h*TYR homology model. The model was based on human tyrosinase-related protein 1 (*h*TRP1, PDB: 5M8Q) which shares 45.81% sequence identity with *h*TYR (Fig. S37 in Supplementary data). Binding interactions between **1b**, **1f**, or kojic and the *h*TYR homology model are shown in Fig. 6a in 2D and 3D.

As shown by the 3D structure, the 2-hydroxymethyl group on the 4-pyrone ring of kojic acid coordinates with the two zinc ions (Zn6 and Zn7) at distances of 2.16 and 2.21 Å, respectively. Kojic acid also forms a hydrogen bond with Asn364 using the 5-hydroxyl group of its 4-pyrone ring and interacts by pi-pi stacking with His367 with a docking score of − 4.9 kcal/mol. This means that the binding affinity of kojic acid for the *h*TYR homology model was similar to that for *m*TYR.

As regards compound **1b**, the 4-hydroxyl group on its phenyl ring coordinated and formed salt bridges with both zinc ions at distances of 2.16 and 2.20 Å, respectively, which were approximately the same as distances observed for kojic acid. Like kojic acid, the 2-hydroxyl group on the phenyl ring of **1b** formed a hydrogen bond with Met374 and interacted by pi-pi stacking with His363 and His367. Additionally, the 2-hydroxyl group on the phenyl ring and the DHIT carbonyl moiety of **1b** interacted hydrophobically with positively charged Lys306, Ser360, His363, Asn364, His367, Ser375, and Gln376 amino acid residues, which might explain the stronger binding of **1b** than kojic acid to *h*TYR. The docking score of **1b** was − 7.0 kcal/mol (Fig. 6b). On the other hand, compound **1f** showed a binding pattern similar to that with *m*TYR. No metal interaction with zinc ions was observed, and only one hydrogen bond was observed between Asn364 and the 3-hydroxyl group on its phenyl ring. Interestingly, the DHIT moiety of **1f** interacted with a negatively charged amino acid (Asp199) as shown in 2D in Fig. 6a. The docking score of **1f** was − 3.9 kcal/mol. In summary, derivatives **1b** and **1f** were both found to bind directly with the *m*TYR and *h*TYR homology models, and docking simulations of both derivatives with the *m*TYR and the *h*TYR homology model were similar.

#### 2.2.4 Cell study

Since **1b** and **1f** exhibited potent TYR inhibitory activity at the enzyme level, we investigated whether they also inhibited TYR at the cellular level using B16F10 murine melanoma cells.

##### 2.2.4.1 Cell cytotoxicity study

Before examining inhibitory effects of compounds **1b** and **1f** on cellular tyrosinase activity, we examined their cytotoxic effects on B16F10 murine cells using the EZ-cytox assay (Daeil Lab Service, Seoul, Korea). After B16F10 cells had been cultured for 24 h, cells were treated with different concentrations (0, 1, 2, 5, 10 or 20 μM) of **1b** or **1f** for 48 h in a humidified atmosphere. Optical densities (ODs) were measured at 450 nm using a microplate reader.

**Fig. 7:**
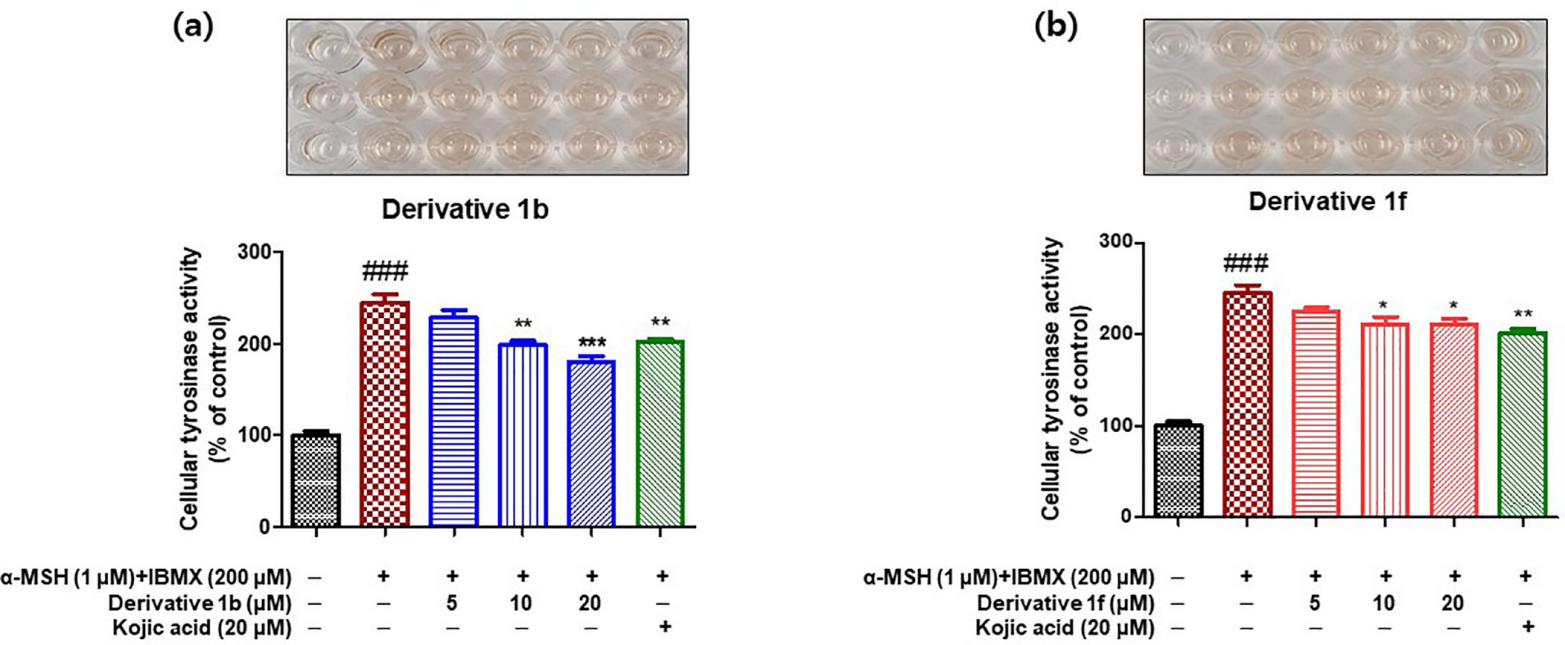
Viabilities of B16F10 cells treated with compounds **1b** (a) or **1f** (b). Cell viability experiments were performed at concentrations of 0, 1, 2, 5, 10, or 20 μM of **1b** or **1f** using an EZ-cytox assay method. Cells were treated with **1b** and **1f** for 48 h and optical densities were measured after treatment with EZ-cytox solution for 1 h. Results are presented as the means ± standard errors of five independent experiments.

Cytotoxicity results are shown in Fig. 7. Neither **1b** nor **1f** had a significant cytotoxic effect on B16F10 cells at concentrations up to 20 μM. Therefore, subsequent cell-based assays on TYR inhibition and intracellular melanogenesis were performed using **1b** and **1f** concentrations of ≤ 20 μM.

##### 2.2.4.2 Cellular tyrosinase inhibition

B16F10 cells co-stimulated with α -melanocyte-stimulating hormone (α -MSH) and 3-isobutyl-1-methylxanthine (IBMX) were used to investigate the inhibitory effects of compounds **1b** and **1f** on mammal cellular tyrosinase. Kojic acid was used as the positive control. After incubation for 24 h, B16F10 melanoma cells were pretreated with 20 μM of kojic acid or derivatives **1b** or **1f** at concentrations of 0, 5, 10, or 20 μM for 1 h and then co-treated with 1 μM of α -MSH and 200 μM of IBMX for 48 h to enhance tyrosinase activity. Cellular tyrosinase activities were determined by measuring optical densities at 492 nm.

**Fig. 8:**
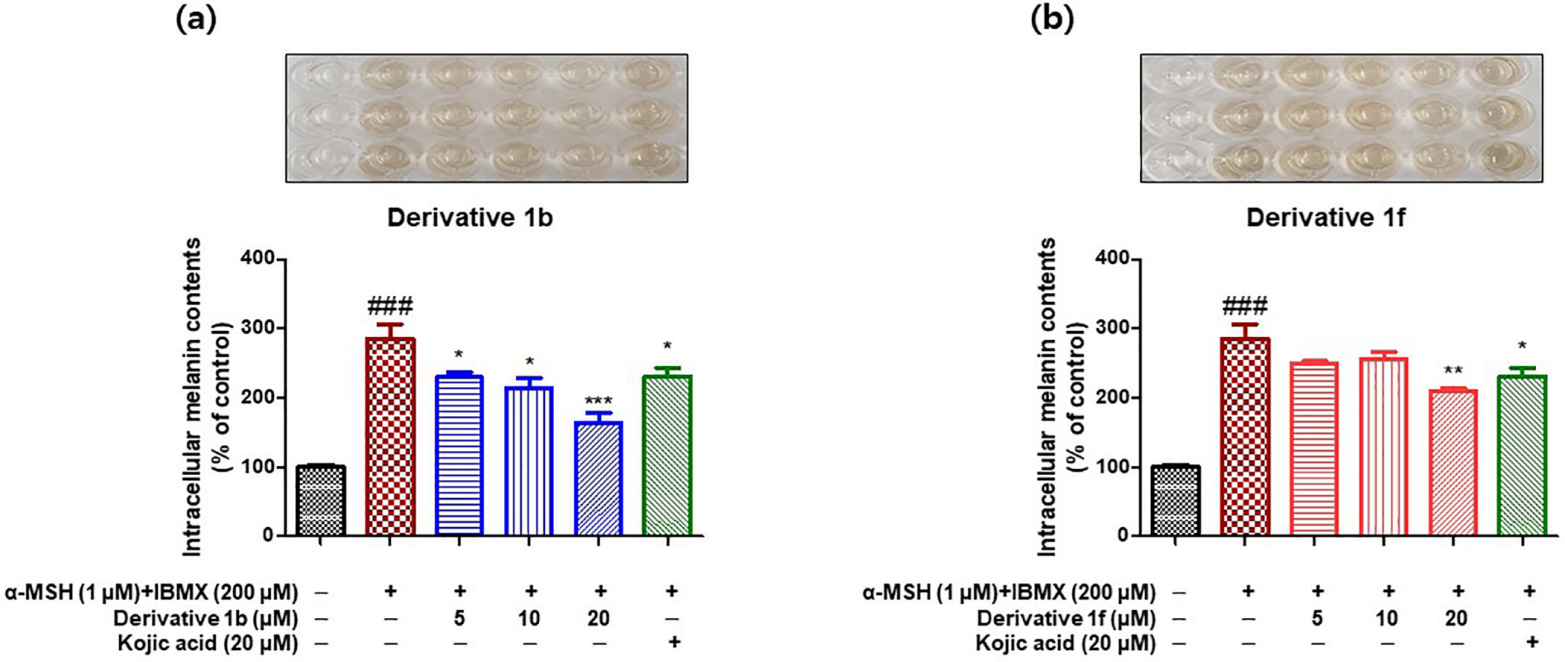
Inhibitory effects of compounds **1b** (a) and **1f** (b) on tyrosinase in B16F10 cells. Cells were stimulated by co-treating them with α -MSH (1 μM) and IBMX (200 μM) and then treated with compounds **1b** or **1f** at 0, 5, 10, or 20 μM, or kojic acid at 20 μM for 48 h. Cellular tyrosinase activities were determined by measuring optical densities at 492 nm. Results are presented as the means ± standard errors of experiments conducted in triplicate. ^###^*P* < 0.001 *vs.* non-treated controls; *P < 0.05, **P < 0.01, ***P < 0.001 *vs.* α -MSH and IBMX-treated cells.

Cellular tyrosinase activity results for derivatives **1b** and **1f** are shown in Fig. 8. Exposure of B16F10 cells to α -MSH and IBMX significantly increased tyrosinase activity to 245% versus non-treated controls and pretreatment with **1b** or **1f** significantly and concentration-dependently reduced this increase in tyrosinase activity. Compound **1b** at 10 μM inhibited cellular TYR to the same extent as compound **1f** or kojic acid at 20 μM.

##### 2.2.4.3 Intracellular melanin inhibition

To determine whether the inhibitory effects of compounds **1b** and **1f** on cellular TYR influence melanogenesis, cellular melanin production was measured. As was performed in TYR inhibition experiments, B16F10 cells co-stimulated with α -MSH and IBMX were used to compare the inhibitory effects of **1b** and **1f** on melanogenesis. Kojic acid was used as the positive control. After incubation for 24 h, B16F10 melanoma cells were pretreated with kojic acid at 20 μM or compounds **1b** or **1f** at 0, 5, 10, or 20 μM for 1 h, and then co-treated with 1 μM of α -MSH and 200 μM of IBMX for 48 h to increase melanin production. Intracellular melanin contents were determined by measuring optical densities at 405 nm.

**Fig. 9:**
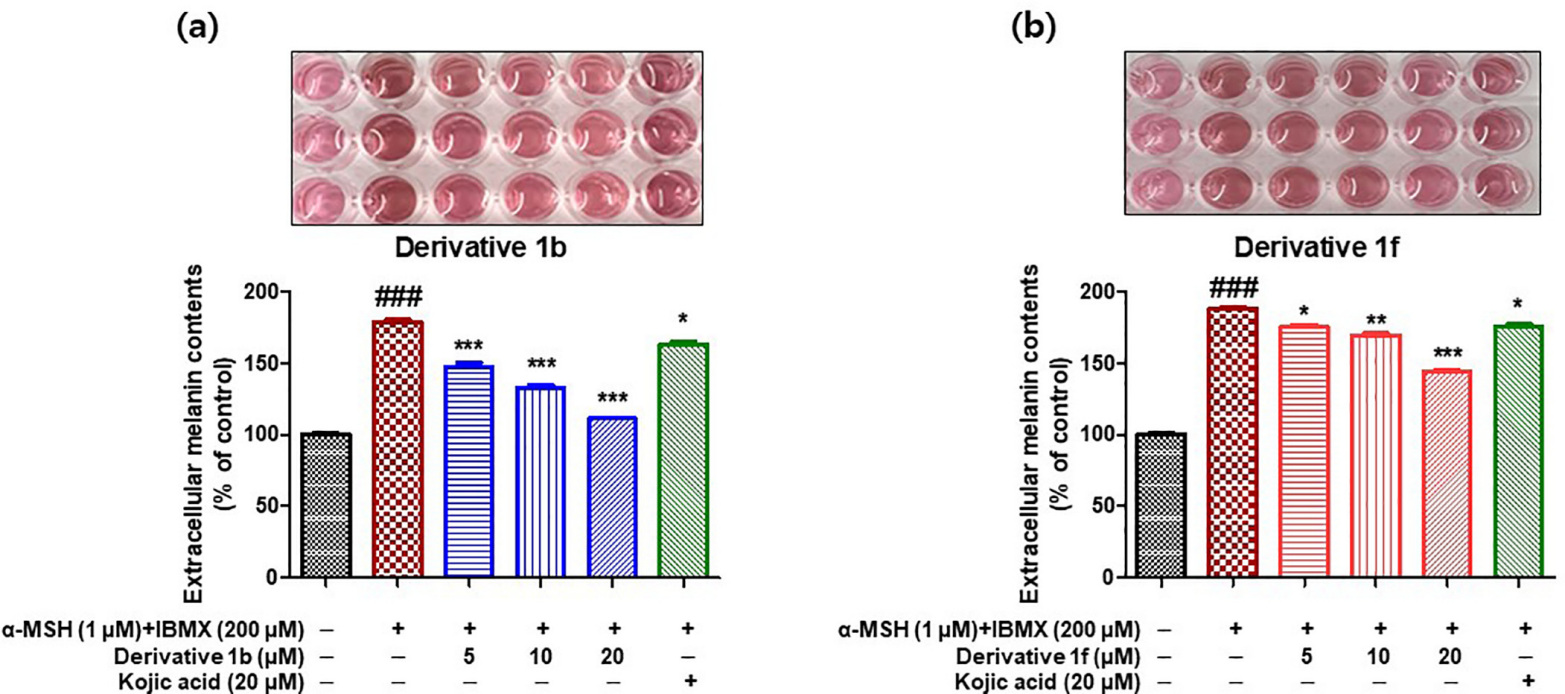
Inhibitory effects of compounds **1b** (a) and **1f** (b) on the melanin contents of B16F10 cells. Cells were stimulated by co-treating them with α -MSH (1 μM) and IBMX (200 μM) and then treated with 0, 5, 10, or 20 μM of **1b** or **1f**, or 20 μM of kojic acid for 48 h. Intracellular melanin contents were determined by measuring optical densities at 405 nm. Results are presented as the means ± standard errors of experiments performed in triplicate. ^###^*P* < 0.001 *vs.* non-treated controls; *P < 0.05, **P < 0.01, ***P < 0.001, *vs.* α -MSH and IBMX-treated cells.

Intracellular melanin content results are provided in Fig. 9. Co-treatment with α -MSH and IBMX increased melanin production to 285% *vs.* non-treated controls but pretreatment with **1b** or **1f** significantly and concentration-dependently suppressed this increase. Compound **1b** at 5 μM suppressed intracellular melanin production by the same extent as kojic acid at 20 μM, and at 20 μM, compound **1f** had a stronger inhibitory effect on melanogenesis than kojic acid.

Furthermore, these inhibitory effects on intracellular melanin contents were similar to those observed on intracellular tyrosinase activity, which indicated the anti-melanogenesis effects of **1b** and **1f** were caused by their TYR inhibitory effects.

##### 2.2.4.4 Inhibition of extracellular melanin levels

To determine whether melanin levels in cell culture media were influenced by compounds **1b** or **1f**, melanin contents in B16F10 cell culture media after treatments with derivatives at different concentrations in the presence of α -MSH and IBMX for 48 h were analyzed by measuring optical densities at 405 nm using a microplate reader.

**Fig. 10:**
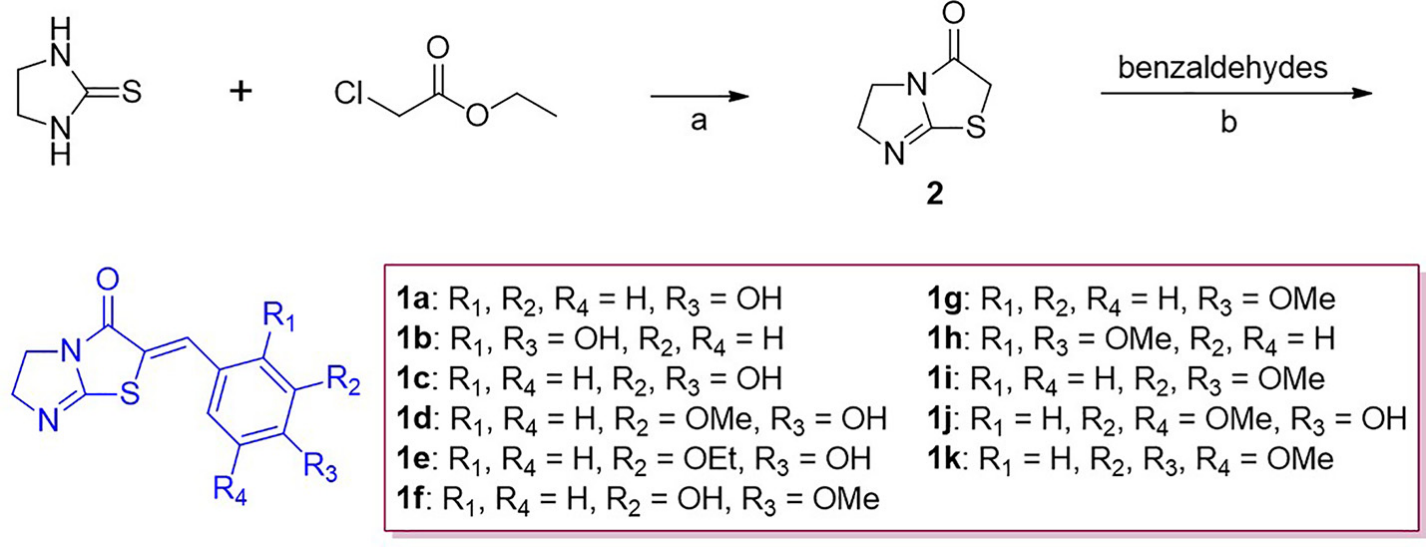
The inhibitory effects of compounds **1b** (a) and **1f** (b) on the release of melanin to medium by B16F10 cells. Cells were stimulated by co-treating them with α -MSH (1 μM) and IBMX (200 μM), and then incubated with compounds **1b** or **1f** at 0, 5, 10, or 20 μM or kojic acid at 20 μM for 48 h. Melanin levels in media were determined by measuring optical densities at 405 nm. Results are presented as the means ± standard errors of experiments performed in triplicate. ^###^*P* < 0.001 *vs.* non-treated controls; *P < 0.05, **P < 0.01, ***P < 0.001 *vs.* α -MSH and IBMX-treated cells.

Melanin levels in media are shown in Fig. 10. These levels were increased by co-treatment with α -MSH and IBMX to 178% *vs.* non-treated controls. However, pretreatment with **1b** or **1f** significantly and concentration-dependently suppressed these increases. Compound **1b** at 5 μM and compound **1f** at 20 μM suppressed these increases more than kojic acid at 20 μM. These results were similar to those obtained for intracellular melanin contents and indicate that **1b** and **1f** both reduced intracellular melanin contents and melanin release to the extracellular compartment.

## 3 Conclusions

Eleven 5,6-dihydroimindazo[2,1-*b*]thiazol-3(2*H*)-one (DHIT) derivatives bearing various benzylidene groups were designed and synthesized as potential tyrosinase inhibitors. Three derivatives (compounds **1a**, **1b**, and **1f**) were found to inhibit mushroom tyrosinase more than kojic acid, and compound **1b** with a 2,4-dihydroxyphenyl ring had an IC_50_ value ∼ 100-fold lower than kojic acid. Kinetic studies demonstrated that compounds **1b** and **1f**, which both inhibited mushroom tyrosinase activity more than kojic acid, were competitive inhibitors. In silico docking simulation results supported that **1b** and **1f** compete with L-tyrosine (a tyrosinase substrate) for the active site of tyrosinase. Cell-based experiments performed on B16F10 melanoma cells demonstrated that compounds **1b** and **1f** suppressed melanogenesis more than kojic acid due to their greater inhibitory effects on cellular tyrosinase. Our *in silico* study conducted using a human tyrosinase homology model supported the notion that **1b** and **1f** are strong candidate anti-melanogenesis agents for the treatment of diseases associated with hyperpigmentation. Furthermore, these results suggest that DHIT might be a useful template for the development of novel and potent tyrosinase inhibitors.

## 4 Materials and methods

### 4.1 Chemistry

#### 4.1.1 General methods

Reagents and chemicals were obtained commercially and used without further purification. All reactions were monitored by thin layer chromatography (TLC) using Merck precoated 60F_245_ plates, and reaction mixtures were purified by column chromatography using MP Silica 40–63 (60 Å). Mass spectroscopy was performed in electrospray ionization (ESI) positive mode using an Expression CMA spectrometer (Advion Ithaca, NY, USA). ^1^H NMR (400 and 500 MHz) and ^13^C NMR (100 and 125 MHz) data were obtained using a Varian Unity INOVA 400 spectrometer or a Varian Unity AS500 spectrometer (Agilent Technologies, Santa Clara, CA, USA). DMSO‑*d*_6_ and CDCl_3_ were used as NMR solvents. Chemical shift values were recorded in parts per million (ppm) and coupling constants (*J*) were recorded in hertz (Hz). The following abbreviations are used: s (singlet), d (doublet), t (triplet), q (quartet), m (multiplet), and brs (broad singlet).

#### 4.1.2 Synthesis of 5,6-dihydroimidazo[2,1-b]thiazol-3(2H)-one (**2**).

To a stirred solution of 2-thioimidazolidine (5.00 g, 48.94 mmol) and sodium acetate (20.07 g, 244.67 mmol) in EtOH (150 mL) was added ethyl chloroacetate (10.47 mL, 97.82 mmol) at 0 °C. The reaction mixture was refluxed for 21 h and cooled, and the precipitate obtained was removed by filtration, washed with EtOH, and the filtrate was partitioned between dichloromethane and water. The organic layer was concentrated *in vacuo* and the resultant residue was purified by silica gel column chromatography using dichloromethane and methanol (20:1) as eluent to give 5,6-dihydroimidazo[2,1-*b*]thiazol-3(2*H*)-one (**2**, 5.00 g) as a white solid (yield 71.9%).

^1^H NMR (400 MHz, CDCl_3_) *δ* 4.33 (t, 2H, *J* = 8.8 Hz, 5-H_2_), 4.12 (s, 2H, 2-H_2_), 3.69 (t, 2H, *J* = 8.8 Hz, 6-H_2_); ^13^C NMR (125 MHz, CDCl_3_) *δ* 164.7, 161.8, 61.8, 41.6, 39.7.

#### 4.1.3 General synthetic procedure for (Z)-2-(substituted benzylidene)-5,6-dihydroimidazo[2,1-b]thiazol-3(2H)-one derivatives (**1a** – **1 k**).

A solution of **2** (100 mg, 0.70 mmol), benzaldehyde (1 equiv.), and NaOAc (173 mg, 2.11 mmol) in acetic acid (1.0 mL) was heated at 80 °C for 2 – 12 h, cooled, and water (5 mL) was added. The precipitates formed were filtered, and washed with water to give **1a** – **1 k** as a solid in yields of 36.9 – 87.5%.

##### 4.1.3.1 (Z)-2-(4-Hydroxybenzylidene)-5,6-dihydroimidazo[2,1-b]thiazol-3(2H)-one (**1a**)

67.9%; ^1^H NMR (500 MHz, DMSO‑*d*_6_) *δ* 10.18 (brs, 1H, OH), 7.55 (s, 1H, vinylic H), 7.43 (d, 2H, *J* = 8.5 Hz, 2′-H, 6′-H), 6.90 (d, 2H, *J* = 8.5 Hz, 3′-H, 5′-H), 4.28 (t, 2H, *J* = 8.5 Hz, 5-H_2_), 3.78 (t, 2H, *J* = 8.5 Hz, 6-H_2_); ^13^C NMR (125 MHz, DMSO‑*d*_6_) *δ* 160.4, 159.7, 157.3, 132.1, 130.4, 124.5, 123.5, 116.7, 61.2, 42.0; LRMS (ESI + ) *m*/*z* 247 (M + H)^+^.

#### 4.1.4 2. (Z)-2-(2,4-Dihydroxybenzylidene)-5,6-dihydroimidazo[2,1-b]thiazol-3(2H)-one (**1b**)

59.4%; ^1^H NMR (500 MHz, DMSO‑*d*_6_) *δ* 10.30 (s, 1H, OH), 10.01 (s, 1H, OH), 7.79 (s, 1H, vinylic H), 7.16 (d, 1H, *J* = 9.0 Hz, 6′-H), 6.39 (s, 1H, 3′-H), 6.37 (d, 1H, *J* = 9.0 Hz, 5′-H), 4.24 (t, 2H, *J* = 8.0 Hz, 5-H_2_), 3.75 (t, 2H, *J* = 8.0 Hz, 6-H_2_); ^13^C NMR (100 MHz, DMSO‑*d*_6_) *δ* 161.6, 161.0, 159.3, 157.8, 129.9, 125.9, 121.8, 112.3, 108.6, 103.2, 61.3, 42.2; LRMS (ESI + ) *m*/*z* 263 (M + H)^+^.

##### 4.1.4.1 (Z)-2-(3,4-Dihydroxybenzylidene)-5,6-dihydroimidazo[2,1-b]thiazol-3(2H)-one (**1c**)

74.2%; ^1^H NMR (400 MHz, DMSO‑*d*_6_) *δ* 9.52 (brs, 2H, 2 × OH), 7.41 (s, 1H, vinylic H), 6.94 (s, 1H, 2′-H), 6.88 (d, 1H, *J* = 8.0 Hz, 6′-H), 6.82 (d, 1H, *J* = 8.0 Hz, 5′-H), 4.22 (t, 2H, *J* = 8.0 Hz, 5-H_2_), 3.73 (t, 2H, *J* = 8.0 Hz, 6-H_2_); ^13^C NMR (100 MHz, DMSO‑*d*_6_) *δ* 160.6, 157.6, 148.6, 146.4, 131.0, 125.1, 123.6, 123.5, 116.9, 116.6, 61.4, 42.2; LRMS (ESI + ) *m*/*z* 263 (M + H)^+^.

##### 4.1.4.2 (Z)-2-(4-Hydroxy-3-methoxybenzylidene)-5,6-dihydroimidazo[2,1-b]thiazol-3(2H)-one (**1d**)

36.9%; ^1^H NMR (400 MHz, DMSO‑*d*_6_) *δ* 9.78 (s, 1H, OH), 7.52 (s, 1H, vinylic H), 7.11 (s, 1H, 2′-H), 7.00 (d, 1H, *J* = 8.0 Hz, 6′-H), 6.88 (d, 1H, *J* = 8.0 Hz, 5′-H), 4.24 (t, 2H, *J* = 8.0 Hz, 5-H_2_), 3.78 (s, 3H, OCH_3_), 3.72 (t, 2H, *J* = 8.0 Hz, 6-H_2_); ^13^C NMR (100 MHz, DMSO‑*d*_6_) *δ* 160.5, 157.5, 149.4, 148.6, 131.0, 125.1, 124.0, 123.7, 116.8, 114.4, 61.5, 56.3, 42.2; LRMS (ESI + ) *m*/*z* 277 (M + H)^+^.

##### 4.1.4.3 (Z)-2-(3-Ethoxy-4-hydroxybenzylidene)-5,6-dihydroimidazo[2,1-b]thiazol-3(2H)-one (**1e**)

71.8%; ^1^H NMR (500 MHz, DMSO‑*d*_6_) *δ* 7.53 (s, 1H, vinylic H), 7.11 (d, 1H, *J* = 1.5 Hz, 2′-H), 7.01 (dd, 1H, *J* = 8.5, 1.5 Hz, 6′-H), 6.91 (d, 1H, *J* = 8.5 Hz, 5′-H), 4.26 (t, 2H, *J* = 8.5 Hz, 5-H_2_), 4.06 (q, 2H, *J* = 7.0 Hz, C*H_2_*CH_3_), 3.77 (t, 2H, *J* = 8.5 Hz, 6-H_2_), 1.34 (t, 3H, *J* = 7.0 Hz, CH_2_C*H_3_*); ^13^C NMR (100 MHz, DMSO‑*d*_6_) *δ* 160.5, 157.5, 149.7, 147.7, 131.0, 125.1, 123.9, 123.8, 116.8, 115.4, 64.5, 61.5, 42.2, 15.3; LRMS (ESI + ) *m*/*z* 291 (M + H)^+^.

##### 4.1.4.4 (Z)-2-(3-Hydroxy-4-methoxybenzylidene)-5,6-dihydroimidazo[2,1-b]thiazol-3(2H)-one (**1f**)

62.9%; ^1^H NMR (500 MHz, DMSO‑*d*_6_) *δ* 9.45 (s, 1H, OH), 7.49 (s, 1H, vinylic H), 7.06 (d, 1H, *J* = 9.0 Hz, 6′-H), 7.04 (d, 1H, *J* = 9.0 Hz, 5′-H), 7.01 (s, 1H, 2′-H), 4.27 (t, 2H, *J* = 8.0 Hz, 5-H_2_), 3.82 (s, 3H, OCH_3_), 3.78 (t, 2H, *J* = 8.0 Hz, 6-H_2_); ^13^C NMR (125 MHz, DMSO‑*d*_6_) *δ* 160.2, 157.2, 149.9, 147.3, 130.4, 126.2, 124.6, 122.9, 116.0, 112.9, 61.2, 56.1, 42.0; LRMS (ESI + ) *m*/*z* 277 (M + H)^+^.

##### 4.1.4.5 (Z)-2-(4-Methoxybenzylidene)-5,6-dihydroimidazo[2,1-b]thiazol-3(2H)-one (**1 g**)

65.2%; ^1^H NMR (500 MHz, CDCl_3_) *δ* 7.64 (s, 1H, vinylic H), 7.44 (d, 2H, *J* = 8.0 Hz, 2′-H, 6′-H), 6.98 (d, 2H, *J* = 8.0 Hz, 3′-H, 5′-H), 4.42 (t, 2H, *J* = 8.0 Hz, 5-H_2_), 3.87 (t, 2H, *J* = 8.0 Hz, 6-H_2_), 3.85 (s, 3H, OCH_3_); ^13^C NMR (125 MHz, CDCl_3_) *δ* 160.9, 160.9, 159.0, 131.5, 131.2, 126.0, 124.3, 114.6, 60.9, 55.4, 41.8; LRMS (ESI + ) *m*/*z* 261 (M + H)^+^.

##### 4.1.4.6 (Z)-2-(2,4-Dimethoxybenzylidene)-5,6-dihydroimidazo[2,1-b]thiazol-3(2H)-one (**1 h**)

72.7%; ^1^H NMR (500 MHz, CDCl_3_) *δ* 7.97 (s, 1H, vinylic H), 7.37 (d, 1H, *J* = 9.0 Hz, 6′-H), 6.56 (dd, 1H, *J* = 9.0, 2.5 Hz, 5′-H), 6.46 (d, 1H, *J* = 2.0 Hz, 3′-H), 4.39 (t, 2H, *J* = 8.5 Hz, 5-H_2_), 3.86 (s, 3H, OCH_3_), 3.85 (t, 2H, *J* = 8.5 Hz, 6-H_2_), 3.84 (s, 3H, OCH_3_); ^13^C NMR (100 MHz, CDCl_3_) *δ* 162.9, 161.4, 159.9, 159.7, 130.3, 127.0, 124.5, 115.8, 105.3, 98.8, 61.0, 55.7, 55.7, 42.1; LRMS (ESI + ) *m*/*z* 291 (M + H)^+^.

##### 4.1.4.7 (Z)-2-(3,4-Dimethoxybenzylidene)-5,6-dihydroimidazo[2,1-b]thiazol-3(2H)-one (**1i**)

87.5%; ^1^H NMR (500 MHz, CDCl_3_) *δ* 7.60 (s, 1H, vinylic H), 7.08 (dd, 1H, *J* = 8.0, 2.0 Hz, 6′-H), 6.97 (d, 1H, *J* = 2.0 Hz, 2′-H), 6.93 (d, 1H, *J* = 8.0 Hz, 5′-H), 4.40 (t, 2H, *J* = 8.5 Hz, 5-H_2_), 3.91 (s, 6H, 2 × OCH_3_), 3.85 (t, 2H, *J* = 8.5 Hz, 6-H_2_); ^13^C NMR (100 MHz, CDCl_3_) *δ* 161.0, 158.8, 150.7, 149.4, 131.4, 126.5, 124.9, 123.7, 112.3, 111.5, 61.3, 56.2, 56.1, 42.0; LRMS (ESI + ) *m*/*z* 291 (M + H)^+^.

##### 4.1.4.8 (Z)-2-(4-Hydroxy-3,5-dimethoxybenzylidene)-5,6-dihydroimidazo[2,1-b]thiazol-3(2H)-one (**1j**)

54.5%; ^1^H NMR (500 MHz, DMSO‑*d*_6_) *δ* 9.20 (brs, 1H, OH), 7.56 (s, 1H, vinylic H), 6.86 (s, 2H, 2′-H, 6′-H), 4.28 (t, 2H, *J* = 8.5 Hz, 5-H_2_), 3.82 (s, 6H, 2 × OCH_3_), 3.79 (t, 2H, *J* = 8.5 Hz, 6-H_2_); ^13^C NMR (100 MHz, DMSO‑*d*_6_) *δ* 160.5, 157.4, 148.9, 138.6, 131.2, 124.4, 124.0, 108.0, 61.5, 56.7, 42.3; LRMS (ESI + ) *m*/*z* 307 (M + H)^+^.

##### 4.1.4.9 (Z)-2-(3,4,5-Trimethoxybenzylidene)-5,6-dihydroimidazo[2,1-b]thiazol-3(2H)-one (**1 k**)

78.2%; ^1^H NMR (400 MHz, CDCl_3_) *δ* 7.58 (s, 1H, vinylic H), 6.69 (s, 2H, 2′-H, 6′-H), 4.41 (t, 2H, *J* = 8.4 Hz, 5-H_2_), 3.88 (s, 6H, 2 × OCH_3_), 3.87 (s, 3H, 4′–OCH_3_), 3.87 (t, 2H, *J* = 8.4 Hz, 6-H_2_); ^13^C NMR (100 MHz, CDCl_3_) *δ* 160.7, 159.0, 153.8, 139.9, 131.8, 129.0, 126.4, 107.1, 61.2, 61.1, 56.4, 42.1; LRMS (ESI + ) *m*/*z* 321 (M + H)^+^.

### 4.2 Tyrosinase inhibition - Kinetics, mechanism, and *in silico* and in vitro studies

#### 4.2.1 Mushroom tyrosinase inhibition assay

The tyrosinase inhibitory activity assays on the synthesized DHIT derivatives **1a** – **1 k** was performed using mushroom tyrosinase (*m*TYR), as previously described [72] with minor modification. Briefly, a 200 μL mixture containing tyrosinase solution (20 μL, 200 units), a DHIT derivative (10 μL, final concentration 25 μM), and substrate solution (170 μL, comprising 14.7 mM potassium phosphate buffer and 293 μM L-tyrosine solution (1:1, v/v)) was added to a 96-well microplate and incubated for 30 min at 37 °C. The optical densities of dopachrome produced during incubation were measured using a microplate reader (VersaMax™, Molecular Devices, Sunnyvale, CA, USA) at 492 nm. Kojic acid (25 μM) was used as the positive control. All experiments were conducted in triplicate. To calculate the tyrosinase inhibitory activities, the following formula was used: % inhibition = [(1− (A/B)) × 100], where A is test sample optical density and B is the optical density of the non-treated control.

To calculate the concentration required to inhibit enzyme activity by 50% (IC_50_), tyrosinase inhibition percentages of each DHIT derivative were obtained at 5 or more different concentrations. IC_50_ values were calculated by plotting linear regression curves of percentage inhibitions versus derivative concentrations. The negative control was obtained by adding dimethyl sulfoxide (DMSO) instead of a DHIT derivative. Kojic acid was used as the positive control.

#### 4.2.2 Kinetics of mTYR inhibitions by **1b** and **1f**

Lineweaver-Burk plots were used to determine modes of *m*TYR inhibition. Compounds **1b** or **1f** (10 μL, final concentrations: 0, 0.0625, 0.125, or 0.25 μM for **1b** and 0, 1, 2 or 4 μM for **1f**) and *m*TYR solution (20 μL, 150 units) were added to a 96-well plate containing 170 μL of a mixture containing aqueous L-tyrosine solution at final concentrations of 0.5, 1.0, 2.0, 4.0, 8.0, or 16 mM for **1b** or 1.0, 2.0, 4.0, or 8.0 mM for **1f**; aqueous L-tyrosine solution, distilled water, and 50 mM potassium phosphate buffer (pH 6.5) in the ratio 10:9:10. Initial rates of dopachrome formations in reaction mixtures were calculated by measuring increases in optical density at 492 nm (ΔOD_492_/min) using a microplate reader (VersaMax^TM^, Molecular Devices, Sunnyvale, CA, USA). Maximum velocities of tyrosinase-L-tyrosine reactions were determined using Lineweaver-Burk plots (inverse of reaction velocity (1/V) versus the inverse of L-tyrosine concentration (1/[S])) obtained using 4 – 5 different concentrations of L-tyrosine. Modes of tyrosinase inhibition were determined using convergence points of plots.

#### 4.2.3 In silico study on interactions between tyrosinase and compounds **1b** or **1f** or kojic acid

##### 4.2.3.1 In silico studies of interactions between mushroom tyrosinase and DHIT derivatives **1b** and **1f**.

Docking studies on compounds **1b** and **1f** and kojic acid were performed using Schrodinger suite (2021–1) as previously described protocols [73] with slight modification. The crystal structure of *m*TYR (PDB ID 2Y9X) was imported from the Protein Data Bank (PDB) using Maestro 12.4 Protein Preparation Wizard and prepared in protein preparation wizard by removing unwanted protein chains. To further refine the structure, hydrogen atoms were added, water molecules > 3 Å from the ligand were removed, and the structure was minimized. In minimized protein structures, glide grid and active sites were determined using the tyrosinase binding site obtained from the PDB and the literature [74], [75], [76]. The structures of **1b**, **1f**, and kojic acid were then imported into the entry list of Maestro in CDXML format. Prior to ligand docking, the structures of **1b**, **1f**, and kojic acid were developed using LigPrep. Compounds were then docked to the glide grid using Glide from the Maestro task list [77]. Binding affinities and ligand–protein interactions were obtained using the glide extra precision (XP) method [78].

##### 4.2.3.2 In silico studies of interactions between the hTYR homology model and **1b**, **1f**, and kojic acid

For *in silico* studies on DHIT derivates **1b** and **1f** an *h*TYR homology model was created using the Swiss model Online Server and Schrodinger Suite (2020–2). The *h*TYR (P14679) protein sequence was imported from the UniProt database and a homology model was developed on the Swiss model online server using the human TRP1 (PDB: 5M8Q) template. The model was further processed using Schrödinger suite and verified using Schrodinger Prime (a homology modeling tool in Schrödinger suite). Compounds **1b** and **1f** and kojic acid were docked to a processed human homology model using a protocol similar to that mentioned for *m*TYR docking.

#### 4.2.4 Cell culture

B16F10 murine melanoma cells were acquired from the American Type Culture Collection (ATCC, Manassas, VA, USA) and cultured in Dulbecco’s modified Eagle’s medium (DMEM), supplemented with 10% fetal bovine serum (FBS) and 1% penicillin–streptomycin in a humidified 5% CO_2_ atmosphere at 37 °C. These cells were used for cell viability, melanin content, and cellular tyrosinase activity assays in 96- or 6-well culture plates.

#### 4.2.5 Cell viability analysis

Cell viability assays were performed at 37 °C as previously described [79] with minor modification using the EZ-cytox assay. In brief, B16F10 melanoma cells were seeded in 96-well plates at a density of 1 × 10^4^ cells/well and incubated in a humidified 5% CO_2_ atmosphere for 24 h at 37 °C. Cells were then treated with compounds **1b** or **1f** at 0, 1, 2, 5, 10, or 20 µM/well for 48 h. EZ-cytox solution (10 µL) (Daeil Lab Service, Seoul, Korea) was then added to each well and incubated for 1 h at 37 °C. Cell viabilities were assessed by measuring absorbances at 450 nm using a microplate reader (VersaMax^TM^, Molecular Devices, Sunnyvale, CA, USA). All experiments were independently conducted three times.

#### 4.2.6 Cellular tyrosinase inhibition assays of compounds **1b** and **1f** in B16F10 cells

Cellular tyrosinase inhibition assays were conducted as previously described [80] with minor modification. B16F10 cells were seeded at a density of 1 × 10^5^ cells/well in 6-well plates and incubated in a humidified 5% CO_2_ atmosphere for 24 h at 37 °C. Cultured B16F10 cells were then treated with **1b**, **1f**, or kojic acid dissolved in DMSO at final concentrations of 0, 5, 10, or 20 µM for **1b** and **1f** or 20 µM for kojic acid for 1 h. α-MSH (final concentration: 1 µM) and IBMX (final concentration: 200 µM) were then added, and cells were incubated in a humidified 5% CO_2_ atmosphere for 48 h at 37 °C. Cells were then washed twice with PBS, lysed by adding 45 mM of phosphate buffer (100 µL) containing 1% Triton X-100 (5 µL) and 1% PMSF (5 µL, phenylmethylsulfonyl fluoride) and frozen at −80 °C for 1 h. Lysates were clarified by centrifugation at 12,000 rpm for 30 min at 4 °C. Cell lysate supernatants (80 µL/well) in a 96-well plate were then mixed with 20 µL of l-DOPA (2 mg/mL in distilled water) and incubated for 30 min at 37 °C. Absorbances of reaction mixtures at 492 nm were recorded using a microplate reader (VersaMax^TM^, Molecular Devices, Sunnyvale, CA, USA). Kojic acid was used as the positive control. Protein concentrations were determined using Bicinchoninic Acid (BCA) protein assay reagent using Bovine Serum Albumin (BSA) as the standard (Thermo Scientific, Rockford, IL, USA). All experiments were independently conducted three times.

#### 4.2.7 Determinations of intra- and extracellular melanin contents in B16F10 melanoma cells

The inhibitory effects of compounds **1b** and **1f** on intracellular melanin contents were investigated as previously described [81]. B16F10 cells were seeded at a density of 1 × 10^5^ cells/well in 6-well plates and incubated in a humidified 5% CO_2_ atmosphere for 24 h at 37 °C. DHIT derivatives were dissolved in DMSO, and cultured cells were pretreated with compound **1b** or **1f** at 0, 5, 10, or 20 µM, or kojic acid at 20 µM for 1 h, 1 µM of α-MSH and 200 µM of IBMX were then added, and cells were incubated in a humidified 5% CO_2_ atmosphere for 48 h at 37 °C. For extracellular melanin contents, the optical density of the cell culture media was directly measured at 405 nm. On the other hand, for intracellular melanin contents, the following procedure was performed. Briefly, after washing twice with PBS, adherent cells were detached by incubation in Trypsin/EDTA for 1 min. Pellets were dissolved in 100 μL of 1 N NaOH and then incubated for 1 h at 60 °C to dissolve the melanin. Intracellular melanin was quantified by measuring optical densities at 405 nm using a microplate reader (VersaMax^TM^, Molecular Devices, Sunnyvale, CA, USA). Results were normalized versus cell pellet total protein using BCA protein assay reagent using BSA as the standard (Thermo Scientific, Rockford, IL, USA). The intracellular melanin contents were calculated using the following equation: (ΔOD_sample_/ΔOD_control_) × 100%; where ΔOD_sample_ = the optical density of the test compound, and ΔOD_control_ = the optical density of control. Experiments were performed in triplicate.

#### 4.2.8 Statistical analysis

Statistical analysis was carried out using GraphPad Prism (La Jolla, CA, USA) and results are presented as means ± standard errors of means (SEMs). One-way analysis of variance (ANOVA) followed by the Bonferroni post hoc test was used to determine the significances of intergroup differences. Statistical significance was accepted for P values < 0.05.

## Declaration of Competing Interest

The authors declare that they have no known competing financial interests or personal relationships that could have appeared to influence the work reported in this paper.
