# Supplementary material for: Identification of (Z)-2-benzylidene-dihydroimidazothiazolone derivatives as tyrosinase inhibitors: Anti-melanogenic effects and in silico studies
Source: Comput Struct Biotechnol J. 2022 Feb 12;20:899–912. doi: 10.1016/j.csbj.2022.02.007 (PMC8861568; doi:10.1016/j.csbj.2022.02.007)
Supplement: Supplementary data 1 [file mmc1.docx]

**Supporting Information**

**For**

**Identification of (Z)-2-benzylidene-dihydroimidazothiazolone derivatives as tyrosinase inhibitors: anti-melanogenic effects and *in* *silico* studies**

Heejeong Choi^a,†^, Il Young Ryu^a,†^, Inkyu Choi^a,†^, Sultan Ullah^b,†^, Hee Jin Jung^a,†^, Yujin Park^a^, YeJi Hwang^a^, Yeongmu Jeong^a^, Sojeong Hong^a^, Pusoon Chun^c^, Hae Young Chung^a^, Hyung Ryong Moon^a,^*

^a^*College of Pharmacy, Pusan National University, Busan 46241, South Korea*

*^b^Department of Molecular Medicine, The Scripps Research Institute, Florida 33458, USA*

*^c^College of Pharmacy and Inje Institute of Pharmaceutical Sciences and Research, Inje University, Gimhae, Gyeongnam 50834, South Korea*

**Contents**

S1. ^1^H NMR spectrum of compound **1a**………………………………………………………4

S2. ^13^C NMR spectrum of compound **1a**……………………………………………………...5

S3. LRMS spectrum of compound **1a**…………………………………..………………..……6

S4. ^1^H NMR spectrum of compound **1b**………………………………………………………7

S5. ^13^C NMR spectrum of compound **1b**……………………………………………………...8

S6. LRMS spectrum of compound **1b**…………………………………..………………..……9

S7. ^1^H NMR spectrum of compound **1c**……………………………………………………...10

S8. ^13^C NMR spectrum of compound **1c**……………………………………………………..11

S9. LRMS spectrum of compound **1c**…………………………………………………..……12

S10. ^1^H NMR spectrum of compound **1d**……………………………………………………13

S11. ^13^C NMR spectrum of compound **1d**…………………………………..……………….14

S12. LRMS spectrum of compound **1d**…………………………………………..……..……15

S13. ^1^H NMR spectrum of compound **1e**……………………………………………..……...16

S14. ^13^C NMR spectrum of compound **1e**……………………………………………………17

S15. LRMS spectrum of compound **1e**……………………………………………..……..…18

S16. ^1^H NMR spectrum of compound **1f**………………………………………………….…19

S17. ^13^C NMR spectrum of compound **1f**……………………………………………………20

S18. LRMS spectrum of compound **1f**………………………………………………….……21

S19. ^1^H NMR spectrum of compound **1g**……………………………………………………22

S20. ^13^C NMR spectrum of compound **1g**………………………………………...…………23

S21. LRMS spectrum of compound **1g**……………………………………………..…….…24

S22. ^1^H NMR spectrum of compound **1h**……………………………………………………25

S23. ^13^C NMR spectrum of compound **1h**…………………………………………………...26

S24. LRMS spectrum of compound **1h**……………………………………..…………..……27

S25. ^1^H NMR spectrum of compound **1i**……………………………………….…...……….28

S26. ^13^C NMR spectrum of compound **1i**……………………………………………………29

S27. LRMS spectrum of compound **1i**………………………………………………..…...…30

S28. ^1^H NMR spectrum of compound **1j**………………………………………………….…31

S29. ^13^C NMR spectrum of compound **1j**……………………………………………………32

S30. Proton coupled ^13^C NMR spectrum of compound **1j**…………………………………...33

S31. LRMS spectrum of compound **1j**………………………………………………….……34

S32. ^1^H NMR spectrum of compound **1k**……………………………………………………35

S33. ^13^C NMR spectrum of compound **1k**…………………………………………...………36

S34. LRMS spectrum of compound **1k**…………………………………………..………......37

S35. ^1^H NMR spectrum of compound **2**…………………………………….…………….…38

S36. ^13^C NMR spectrum of compound **2**……………………………………….……………39

S37. Homology modeling of human tyrosinase based on human tyrosinase related protein 1 (*h*TRP)………………………………………………………………………………………40

S38. Raw data for IC_50_ calculation…………………………………………………………41

S1. ^1^H NMR spectrum of compound **1a**

S2. ^13^C NMR spectrum of compound **1a**

**
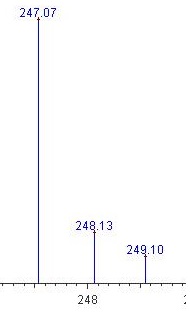
**


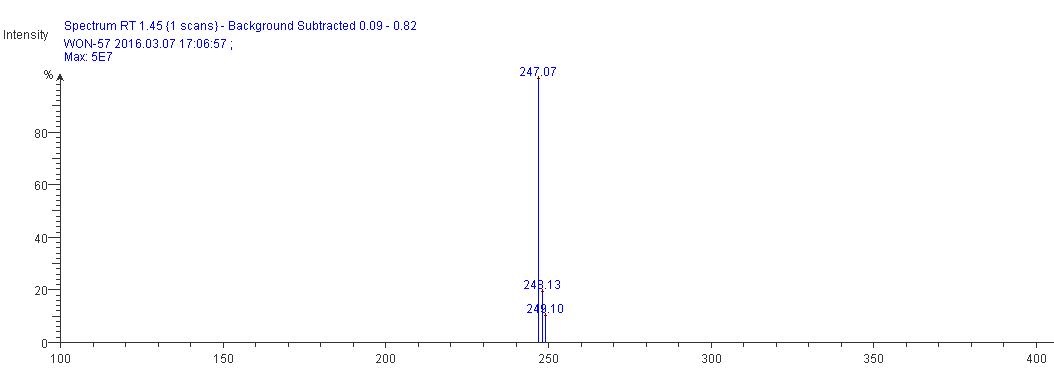


S3. LRMS spectrum of compound **1a**

S4. ^1^H NMR spectrum of compound **1b**

S5. ^13^C NMR spectrum of compound **1b**


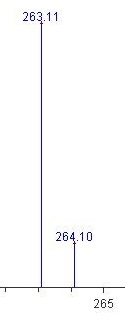

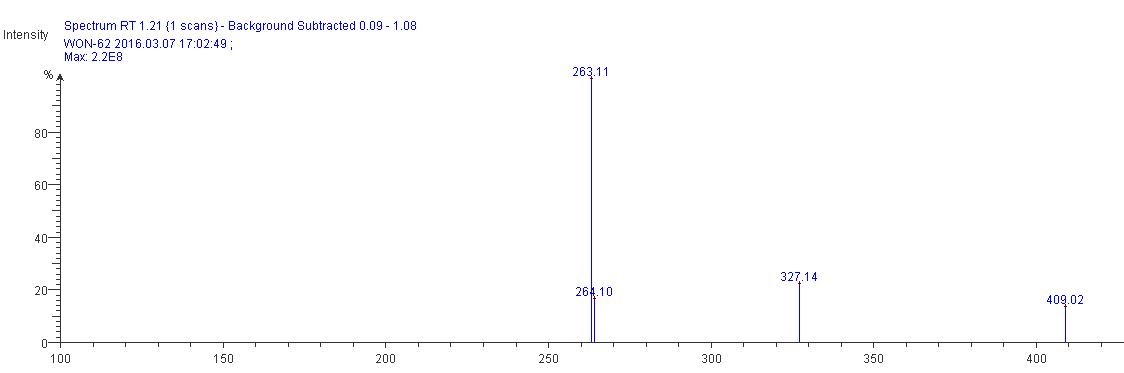


S6. LRMS spectrum of compound **1b**


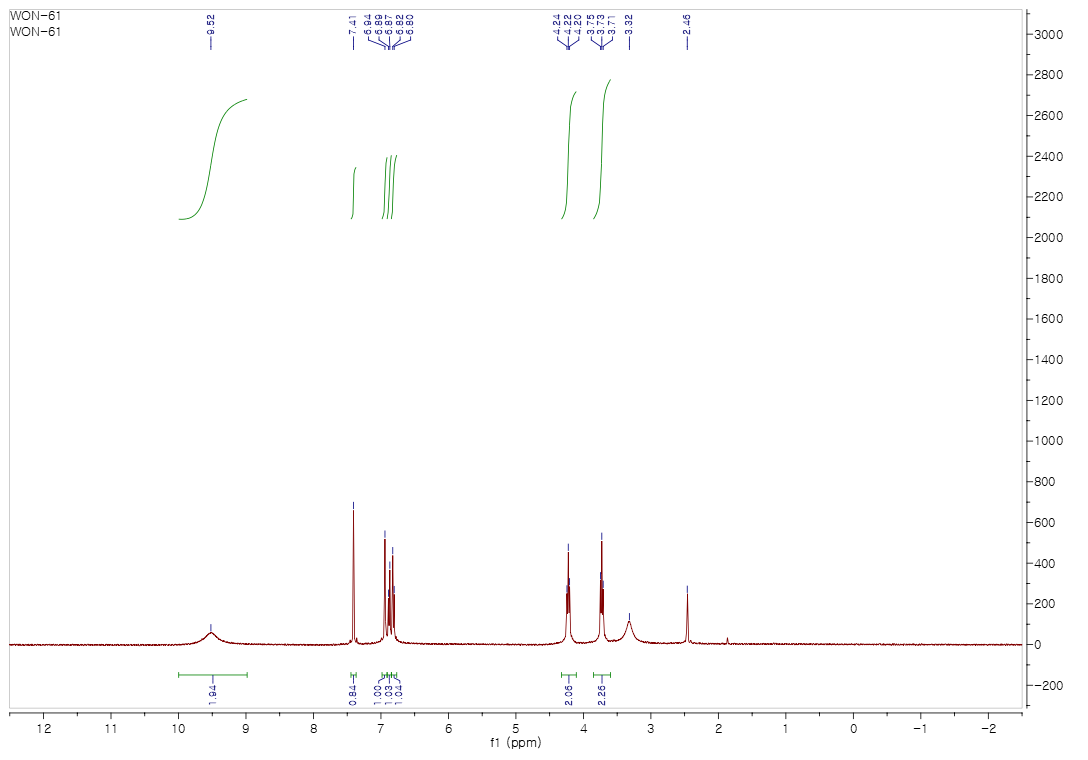


S7. ^1^H NMR spectrum of compound **1c**

S8. ^13^C NMR spectrum of compound **1c**


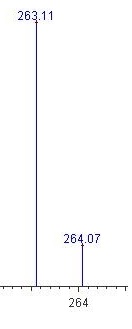

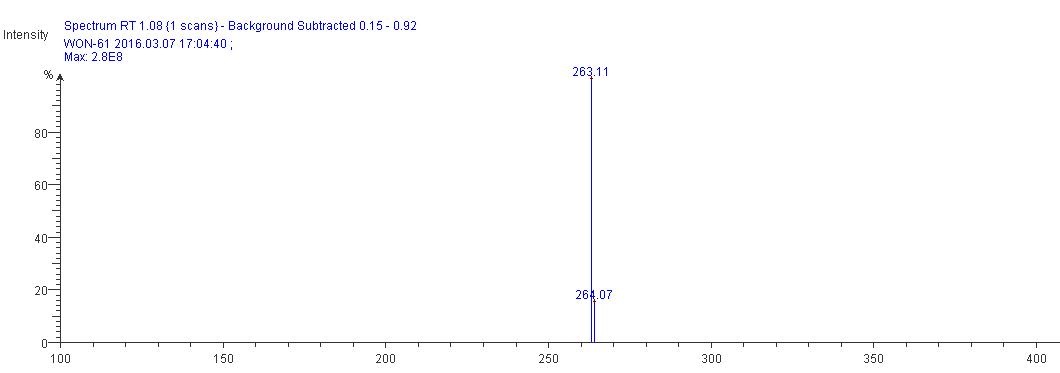


S9. LRMS spectrum of compound **1c**


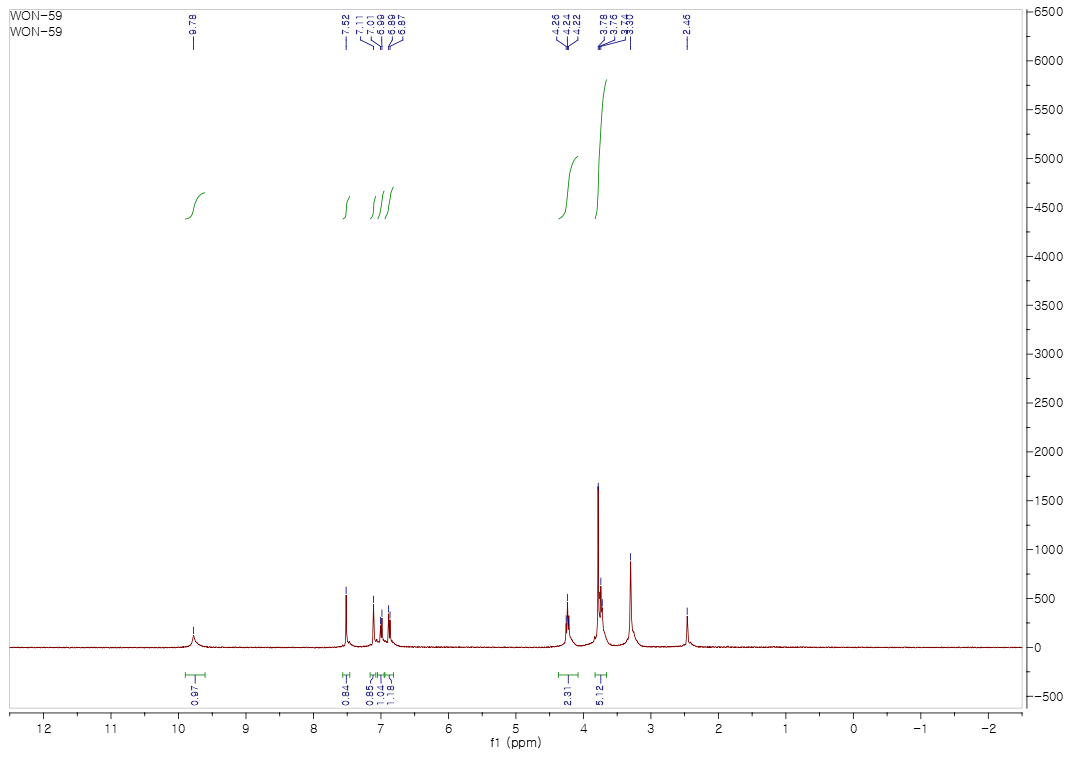


S10. ^1^H NMR spectrum of compound **1d**

S11. ^13^C NMR spectrum of compound **1d**


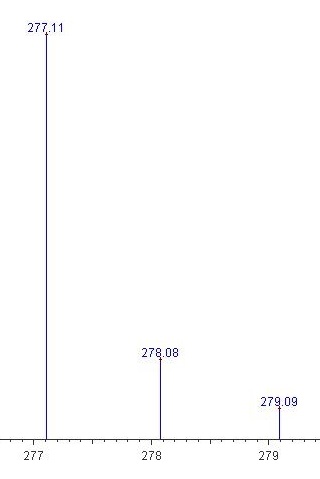

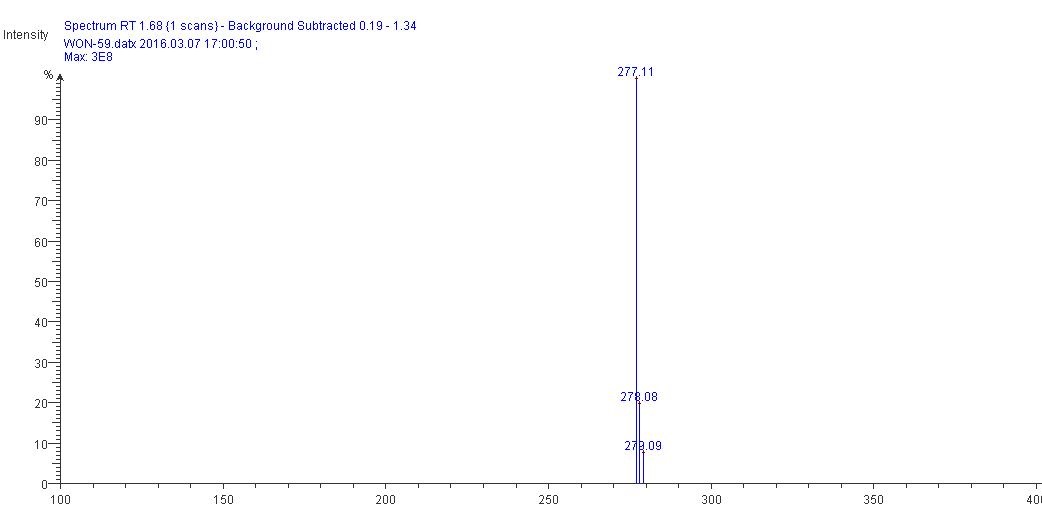


S12. LRMS spectrum of compound **1d**

S13. ^1^H NMR spectrum of compound **1e**

S14. ^13^C NMR spectrum of compound **1e**


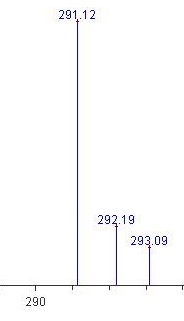

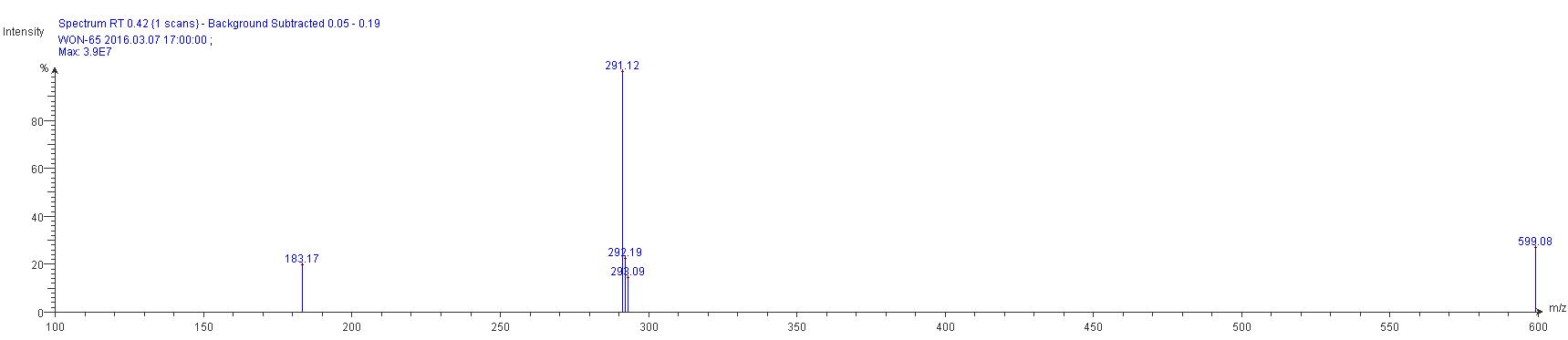


S15. LRMS spectrum of compound **1e**

S16. ^1^H NMR spectrum of compound **1f**

S17. ^13^C NMR spectrum of compound **1f**


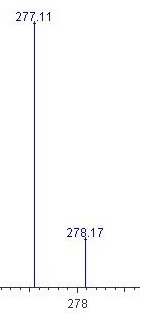

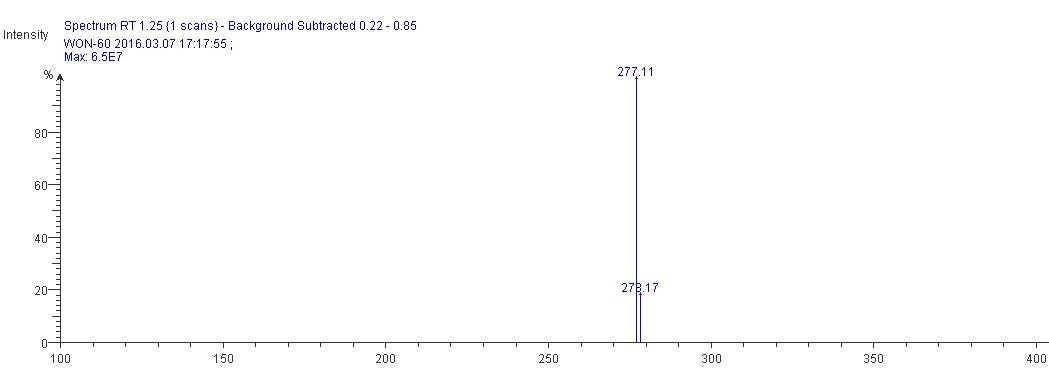


S18. LRMS spectrum of compound **1f**

S19. ^1^H NMR spectrum of compound **1g**

S20. ^13^C NMR spectrum of compound **1g**


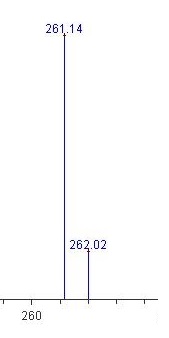

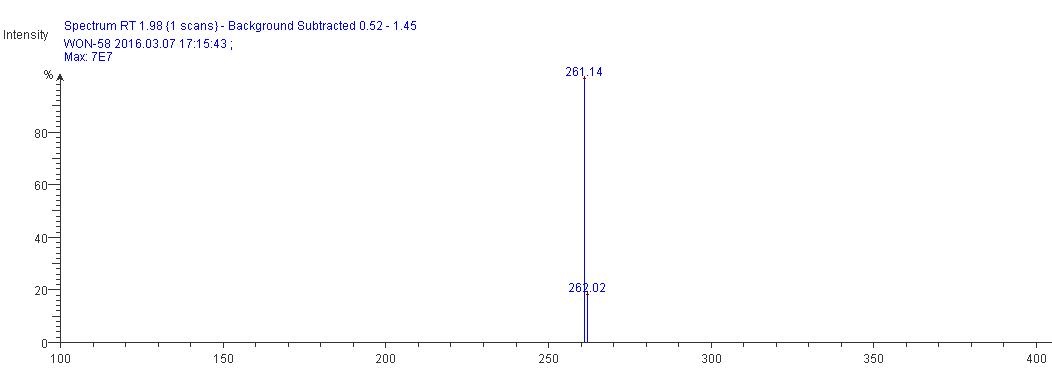


S21. LRMS spectrum of compound **1g**

S22. ^1^H NMR spectrum of compound **1h**

S23. ^13^C NMR spectrum of compound **1h**


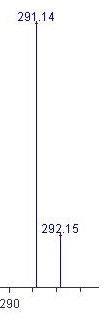

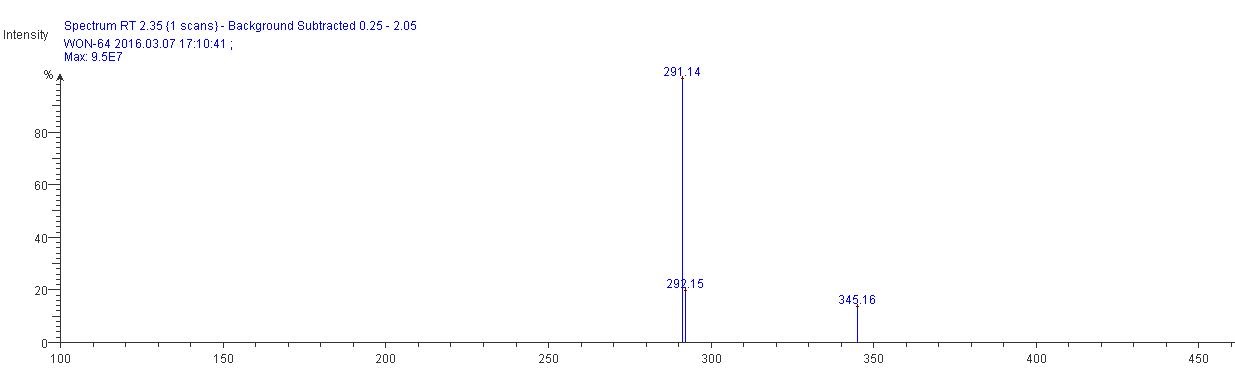


S24. LRMS spectrum of compound **1h**

S25. ^1^H NMR spectrum of compound **1i**

S26. ^13^C NMR spectrum of compound **1i**


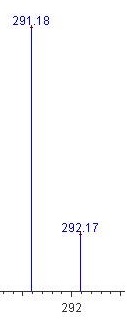

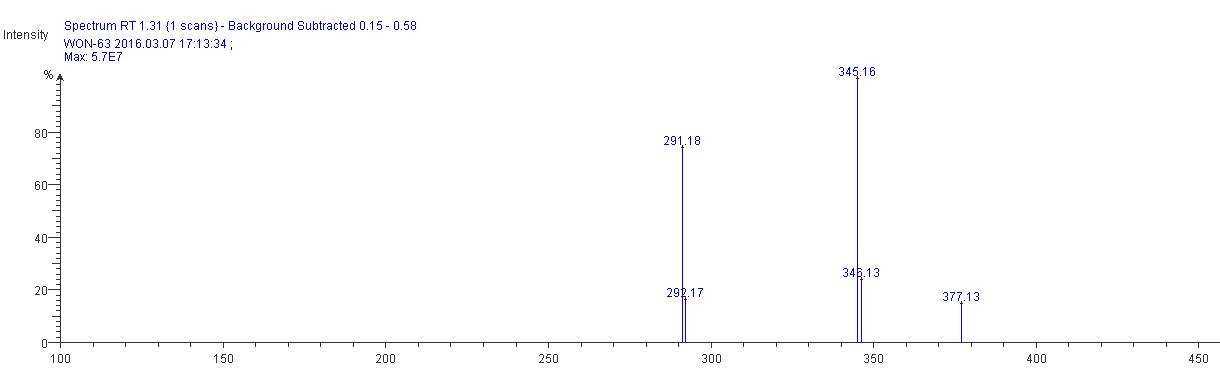


S27. LRMS spectrum of compound **1i**

S28. ^1^H NMR spectrum of compound **1j**

S29. ^13^C NMR spectrum of compound **1j**

S30. Proton coupled ^13^C NMR spectrum of compound **1j** (125 MHz).


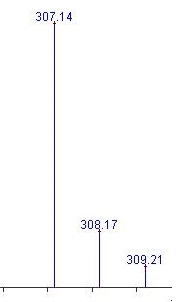

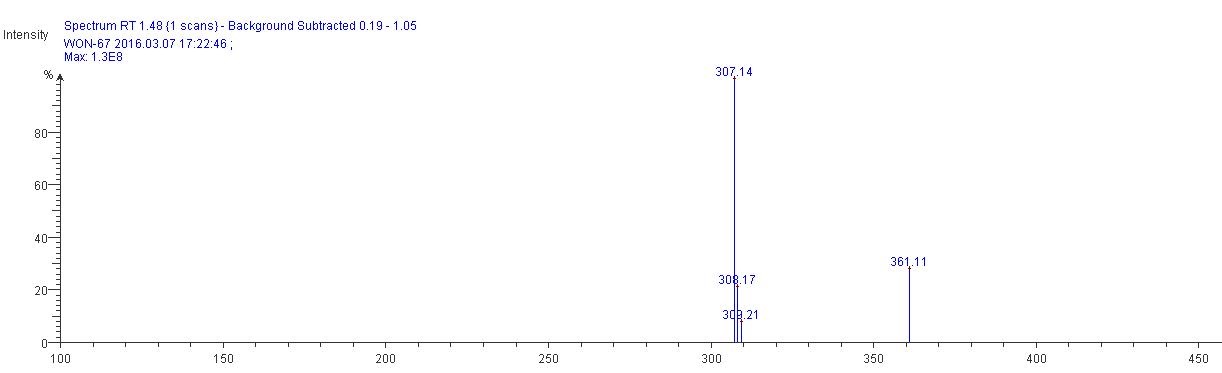


S31. LRMS spectrum of compound **1j**


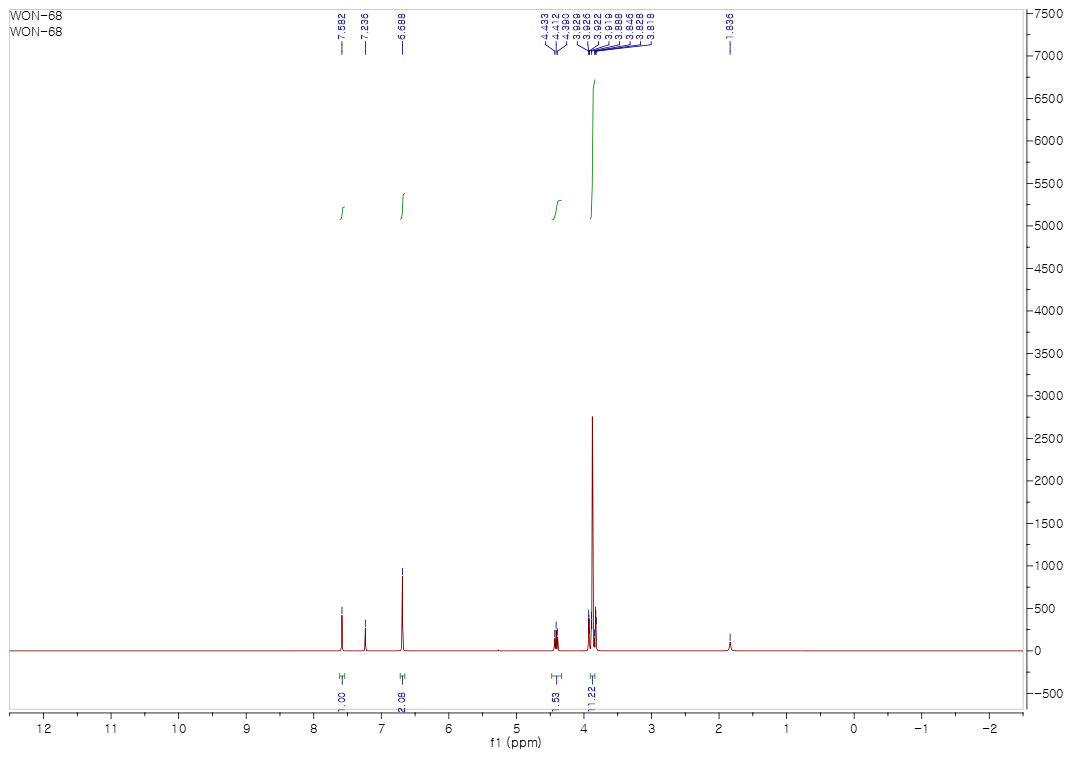


S32. ^1^H NMR spectrum of compound **1k**

S33. ^13^C NMR spectrum of compound **1k**


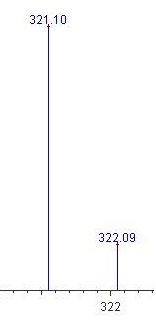

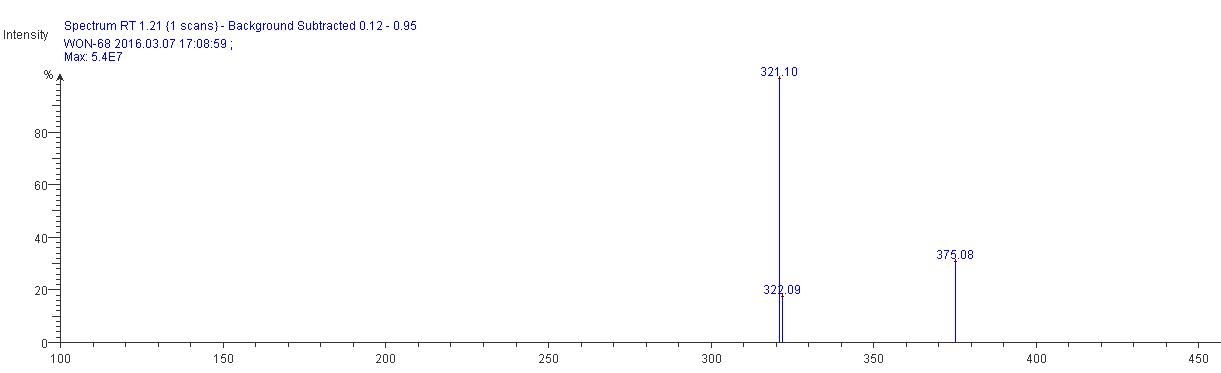


S34. LRMS spectrum of compound **1k**

S35. ^1^H NMR spectrum of compound **2**

S36. ^13^C NMR spectrum of compound **2**


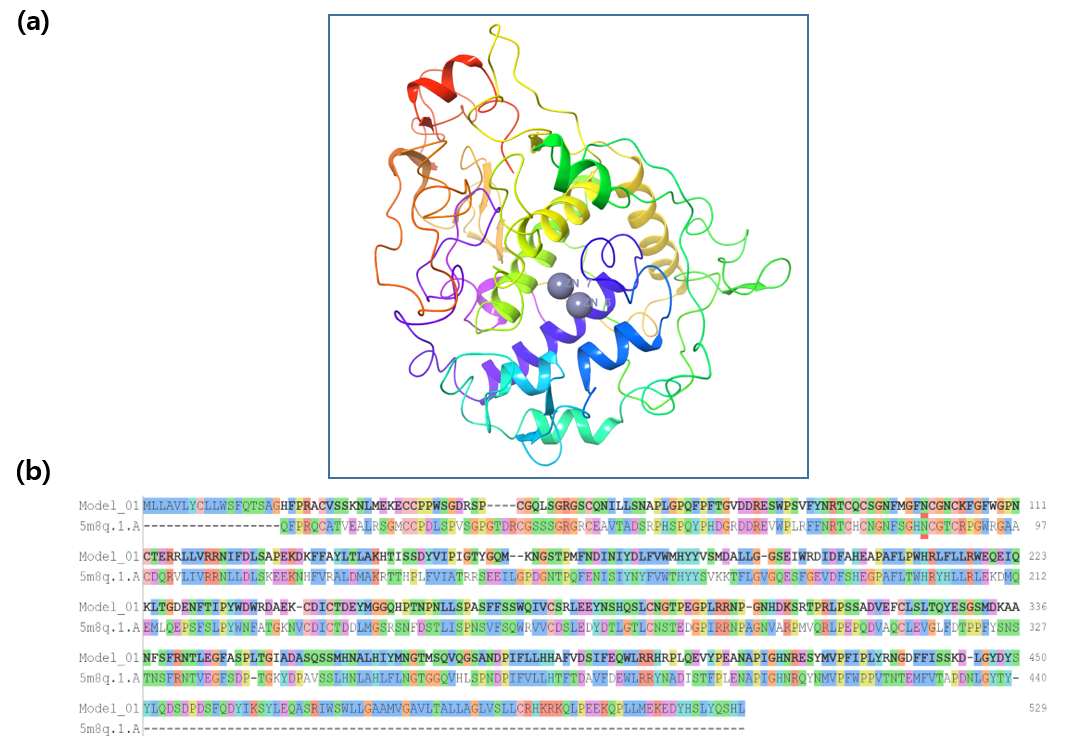


S37. Homology modeling of human tyrosinase based on human tyrosinase related protein 1 (*h*TRP). *(a) Protein structure of human tyrosinase homology model and (b) aligned protein sequences of human tyrosinase homology model and a human TRP1 protein template (5M8Q).*


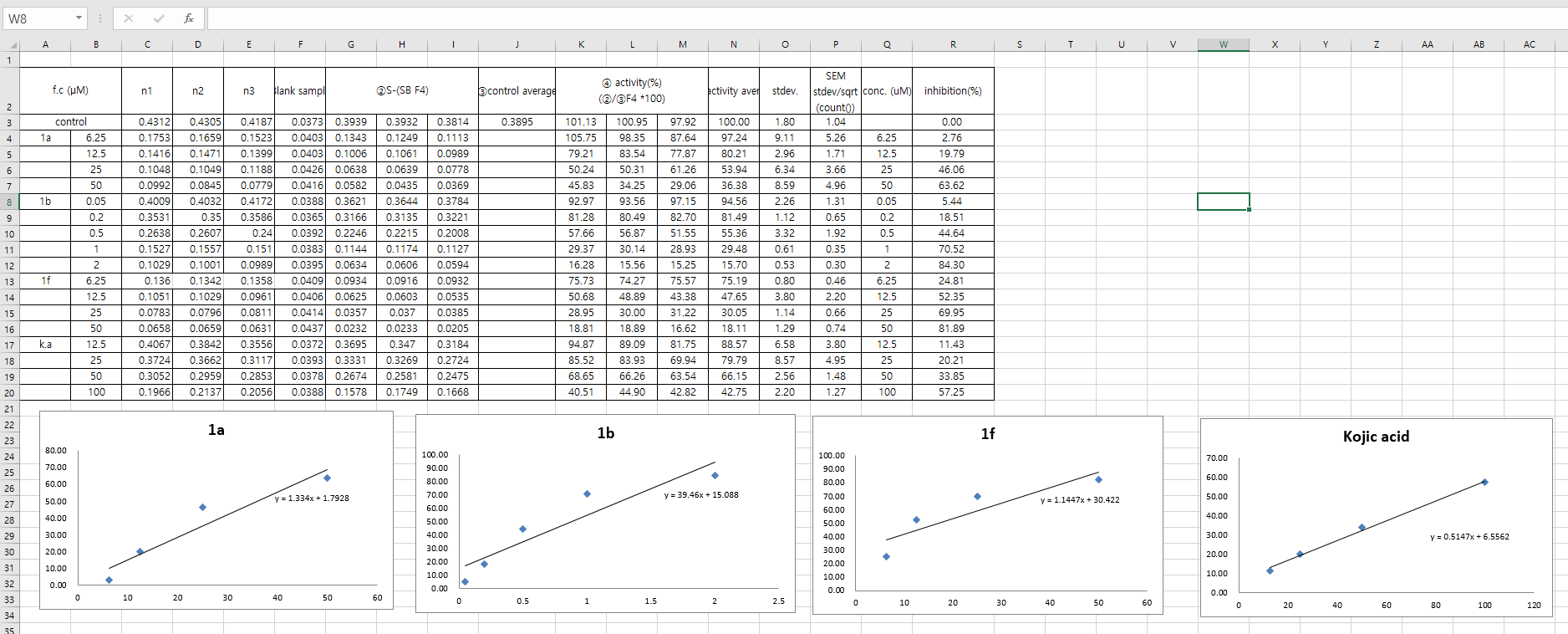


S38. Raw data for IC_50_ calculation.
